# Aerobic catabolism of isobutylene by *Mycolicibacterium* sp. ELW1 requires inducible, plasmid-borne *ibc* genes

**DOI:** 10.1128/aem.02471-25

**Published:** 2026-04-17

**Authors:** John B. Joyce, Nicholas W. Faulkner, Michael R. Hyman, Eric S. Miller

**Affiliations:** 1 Department of Plant and Microbial Biology, North Carolina State University 242504, Raleigh, North Carolina, USA; Shanghai Jiao Tong University, Shanghai, China

**Keywords:** isobutylene, megaplasmid, RNA-seq, proteomics, MTBE, activity-based labeling, monooxygenase

## Abstract

Isobutylene (2-methylpropene [IB]) is the simplest branched alkene and is used industrially to produce a wide range of products, ranging from fuel oxygenates to automobile tires. Only members of the *Mycobacteriaceae* family, including *Mycolicibacterium* sp. ELW1, are known to aerobically catabolize IB as a sole carbon and energy source. A multi-omics approach established two gene clusters on a large catabolic plasmid, pELW1-1, carried by *Mycolicibacterium* sp. ELW1. These two clusters separately contain the predicted genes necessary for the catabolism of IB to 2-hydroxyisobutyrate (2-HIBA) (cluster 1) and the biosynthesis of cobalamin and the further cobalamin-dependent transformation of 2-HIBA to acetyl-CoA (cluster 2). Curing strain ELW1 of pELW1-1 resulted in the loss of the ability of ELW1 to grow on IB and downstream metabolites, including isobutylene oxide (IBO), 2-methyl-1,2-propanediol (MPD), and 2-hydroxyisobutyrate (2-HIBA). Transcriptomic and proteomic analyses, combined with activity-based labeling, growth, and whole-cell activity assays, confirmed that the plasmid-borne *ibc* genes are strongly induced during growth on IB and that the plasmid is obligatory for IB catabolism.

**IMPORTANCE:**

*Mycolicibacterium* sp. ELW1 is one of three strains isolated from across the world that are known to catabolize and grow aerobically on isobutylene (IB), the shortest branched alkene. IB catabolism is initiated by a monooxygenase and proceeds through a pathway that intersects with the methyl *tertiary*-butyl ether (MTBE) biodegradation pathway. The plasmid localization, gene organization, and gene induction pattern shown here provide insights into the role of large catabolic plasmids in the aerobic catabolism of gaseous alkenes.

## INTRODUCTION

Isobutylene (2-methylpropene [IB]) is the simplest branched alkene and is widely used as a feedstock for the industrial production of fuel oxygenates, such as methyl *tertiary*-butyl ether (MTBE), the gasoline blending component, isooctane (2,2,4-trimethylpentane), and butyl rubber, a copolymer with isoprene, which is used to make tires (1, 2). To manufacture these important transportation-related products, >2 million metric tons of IB are produced annually in the United States alone (EPA 2020 CDR Data) (3). Without any substantial biogenic sources, IB is directly extracted from gaseous hydrocarbon mixtures using sulfuric acid, or it can be indirectly produced by the dehydration of *tertiary*-butyl alcohol (TBA) (4).

**Fig 1:**
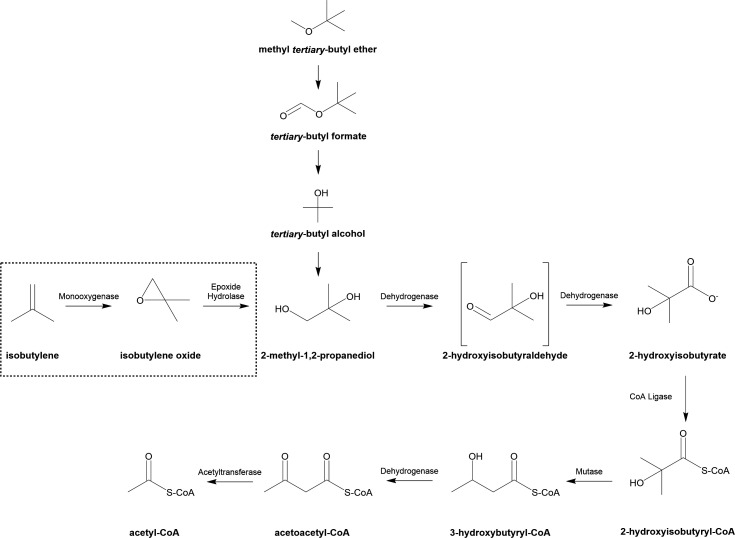
The putative IB/MTBE catabolic pathway in IB-utilizing *Mycobacteriaceae*. Isobutylene transformation (dashed box) in IB-utilizing *Mycobacteriaceae* merges with the well-characterized MTBE biodegradation pathway at the metabolite 2-methyl-1,2-propanediol. Figure adapted from Kottegoda et al. (6), Helbich et al. (5), and Lopes Ferreira et al. (7).

Currently, the only microorganisms known to utilize IB aerobically as a sole carbon and energy source are members of *Mycobacteriaceae* (5). The first aerobic IB-utilizing strain described was *Mycolicibacterium* sp. ELW1 (formerly *Mycobacterium* sp. ELW1, hereafter ELW1). This strain was isolated from the sediment of a freshwater stream in North Carolina. Physiological approaches, including substrate-specific growth, metabolite analyses, and selective inhibitors, were used to develop a pathway of IB catabolism (6) (Fig. 1).

The isobutylene catabolism (Ibc) pathway indicates that IB catabolism by ELW1 is initiated by a monooxygenase that generates an epoxide, 2-methyl-1,2-epoxypropane (isobutylene oxide [IBO]), as the immediate oxidation product. While epoxide formation is a consistent feature of aerobic bacterial alkene oxidation, unlike other alkene-oxidizing bacteria that often conjugate reactive epoxide metabolites with either glutathione or coenzyme M (CoM) (8–11), ELW1 uses hydrolysis to convert IBO to 2-methyl-1,2-propanediol (MPD). In support of this, an epoxide hydrolase from ELW1 has recently been cloned, heterologously expressed, and shown to catalyze this reaction at physiologically relevant rates (12). Following the hydrolysis of IBO to MPD, the Ibc pathway then merges with the MTBE/TBA degradation pathway that has been well-characterized in several other bacterial species (6, 7, 13–15). In this pathway, MPD is sequentially oxidized to 2-hydroxyisobutyraldehyde by an alcohol dehydrogenase and to 2-hydroxyisobutyrate (2-HIBA) by an aldehyde dehydrogenase. Although the predicted aldehyde intermediate (2-hydroxyisobutyraldehyde, 2-HIBAL) has not been detected in any studies reported to date.

There are several potential pathways for the assimilation of 2-HIBA (14, 16), but both IB- and many MTBE-metabolizing bacteria appear to use the pathway established for the versatile gasoline oxygenate-metabolizing bacterium, *Aquincola tertiaricarbonis* L108 (14). In this strain, 2-HIBA undergoes thioesterification with coenzyme A (CoA) to form 2-HIBA-CoA (17), which is then isomerized to 3-hydroxybutyryl-CoA by a cobalamin-dependent acyl-CoA mutase (14, 18–20). This 2-HIBA-CoA mutase has been extensively characterized in relevant MTBE-degrading species (18, 21, 22). In support of this pathway, it has been shown that ELW1 grows well on IB, IBO, MPD, 2-HIBA, and 3-hydroxybutyrate (3HB) in media containing Co^2+^. In contrast, 3HB is the only one of these substrates that supports growth if Co^2+^ is excluded from the growth medium (6), and this is likely because cobalamin-dependent enzymes are not necessary for the catabolism of 3HB. The final steps in IB catabolism likely involve the formation of acetyl-CoA and further metabolism through the central metabolic pathways, possibly utilizing the glyoxylate shunt (23).

Recently, two newly identified aerobic IB-catabolizing bacteria, *Mycolicibacterium gadium* IBE100 (hereafter IBE100) and *Mycobacterium paragordonae* IBE200 (hereafter IBE200), have been described (5). Both strains were isolated from activated sludge from a wastewater treatment plant. Draft genomes containing 87 (IBE100) and 262 (IBE200) contigs were obtained for these microorganisms, and a hybrid transcriptomic analysis of IBE200 with a proteomic analysis of IBE100 was used to assign genes to the enzymes involved in IB catabolism. While this recent molecular analysis retained all of the key reactions and metabolites of the epoxide-generating pathway established for strain ELW1 (6) (Fig. 1), the lack of finished genomes for these two new strains prevented the determination of whether their *ibc* clusters, like many other hydrocarbon-utilizing bacteria, are plasmid-borne (24–26).

Large plasmids that harbor genes encoding partial or full hydrocarbon catabolic pathways are frequently present in aerobic alkene-metabolizing bacteria (11, 27–29). Plasmid loss can result in altered host metabolic profiles and the loss of degradation capacity for specific hydrocarbons and other substrates (30–32). In the case of the model isoprene-catabolizing strain *Rhodococcus* sp. AD45, a 300 Kbp circular plasmid encodes an isoprene-oxidizing monooxygenase (IsoMO) and a transferase (IsoI) that conjugates glutathione with the immediate epoxide product of isoprene oxidation (27). The plasmid also encodes a dehydrogenase (IsoH) that further oxidizes this epoxide-glutathione conjugate (27). Similarly, the well-characterized propylene-oxidizing strain *Xanthobacter autotrophicus* Py2 possesses a 320 Kbp linear plasmid that encodes the propylene-oxidizing monooxygenase (Xamo) that initiates propylene catabolism (30). Other plasmid-borne genes for this bacterium encode enzymes that catalyze additional key processes relevant to propylene catabolism, including epoxide carboxylation and Co-M biosynthesis (30).

In this study, we have resolved the full genome of *Mycolicibacterium* sp. ELW1 and identified two IB-inducible gene clusters on a 221 Kbp circular plasmid, pELW1-1. Physiological studies with a wild-type and a plasmid-cured strain of ELW1 demonstrate that pELW1-1 is essential for growth on IB, and RNA-seq analyses demonstrate that the proposed *ibc* genes are expressed at high levels in response to IB. The same expression trends were also confirmed using shotgun proteomic analyses, whole-cell activity assays, and activity-based labeling (ABL) of the oxygenase component of the IB-oxidizing monooxygenase. Our results have been interpreted in terms of their impact on our understanding of the pathways of bacterial aerobic gaseous alkene catabolism and the role of large plasmids in this process.

## RESULTS AND DISCUSSION

### *Mycolicibacterium* sp. strain ELW1 genome, plasmid, and *ibc* genes

**Fig 2:**
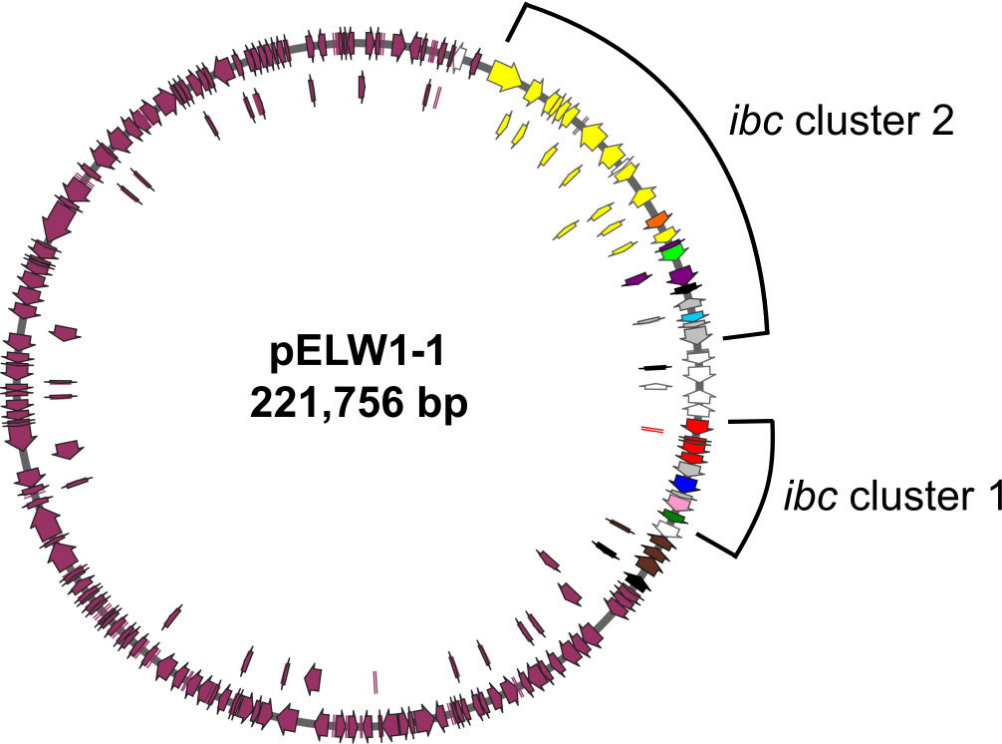
pELW1-1 of *Mycolicibacterium* sp. strain ELW1. The plasmid pELW1-1 is presented highlighting two IB-related clusters: *ibc* cluster 1 and *ibc* cluster 2. Genes from cluster 1 and cluster 2 are color-coded to match Fig. 3, Fig. 5, Tables S2 and S3, and Fig. S2 and S4. Arrows indicate gene orientations, with inner circles of genes placed solely for figure sizing. Figure generated by SnapGene software (https://www.snapgene.com/).

In this study, the complete genome of ELW1 was sequenced twice: once in 2019 using PacBio sequencing and once in 2024 using a hybrid of Oxford Nanopore and Illumina sequencing. The consensus ELW1 genome consisted of a ~6 Mbp chromosome and a single circular ~221 Kbp megaplasmid (pELW1-1) (Fig. 2). CheckM genome analysis revealed 96%–100% completeness (Table S1). The chromosome had an average GC content of 67%, while the plasmid had an average GC content of 66%. The genome for the plasmid-free strain, ELW1ΔpELW1-1, was also sequenced in 2024 using the hybrid Oxford Nanopore and Illumina approach. Sequencing of this strain resulted in a single chromosome with no plasmid. Genome assembly statistics and information for each sequencing approach are shown in Table S1. Minor discrepancies between sequencing data were likely due to sequencing platforms, assembly methods, and annotation tools. Full genome alignments of all three sequencing results yielded nearly identical pairwise alignments based on seed matches using a HOXD scoring matrix (33). The ELW1 genome aligns closely with *Mycolicibacterium aichiense* strain DSM 44147 (92.83% ANI) using the online Microbial Genomes Atlas taxonomy server (34). The ELW1 genome alignments from all sequencing results are depicted in Fig. S1.

### *ibc* cluster 1

**Fig 3:**
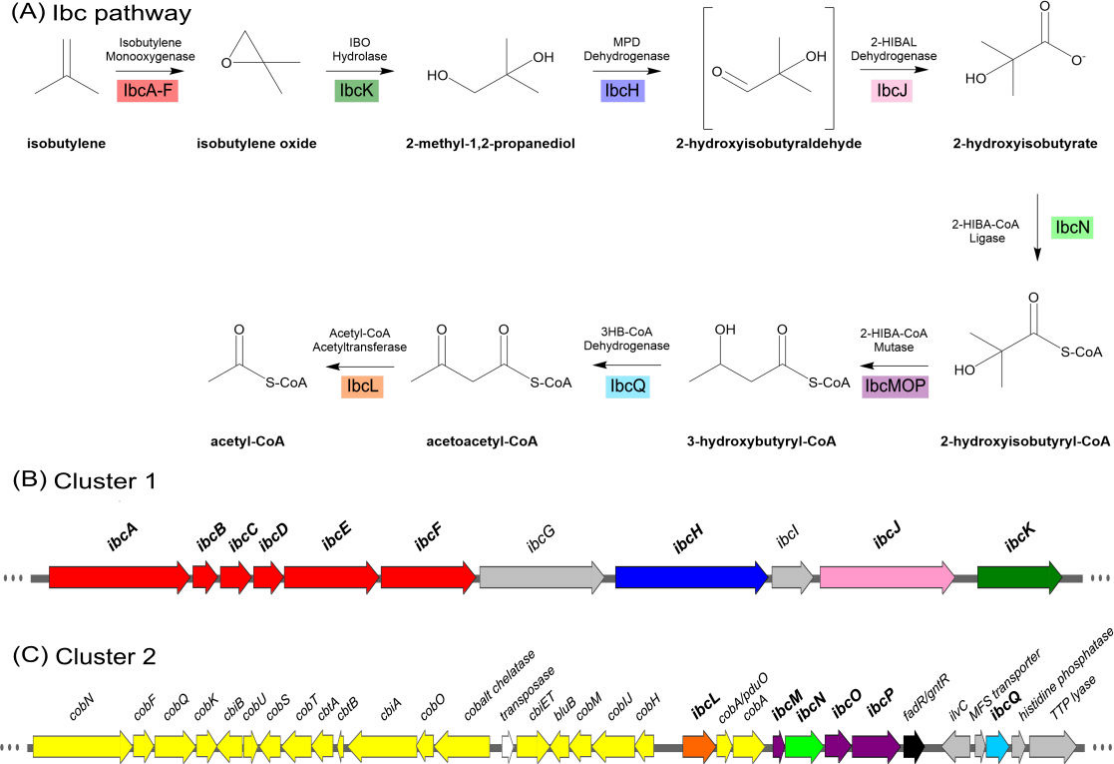
Predicted IB catabolic pathway and pELW1-1 gene clusters in ELW1. Graphical representation of the pathway for IB catabolism and the gene organization of pELW1-1 *ibc* clusters 1 and 2. (**A**) IB catabolic pathway. (**B**) The organization of cluster 1 and the putative genes involved in the degradation of IB to 2-HIBA. Red: subunits for IMO. Dark green: epoxide hydrolase. Dark blue: alcohol dehydrogenase. Pink: aldehyde dehydrogenase. Gray: genes with unknown or unexpected involvement in the previously described pathway. (**C**) The organization of cluster 2 and the putative genes involved in the degradation of 2-HIBA to acetyl-CoA. Light green: CoA ligase. Purple: cobalamin-dependent mutase. Light blue: dehydrogenase. Orange: acetyltransferase. Yellow: cobalamin biosynthesis genes. Gray: genes with unknown or unexpected involvement in the previously described pathway. Black: predicted transcription factor/regulatory genes. White: transposase gene. Color coding corresponds to Fig. 2, Fig. 5, Tables S2 and S3, and Fig. S2 and S4.

Genes encoding proteins involved in the presumptive Ibc pathway (Fig. 3A) were found within two clusters on the megaplasmid pELW1-1 (5, 6). Cluster 1 is a single operon predicted using Operon-Mapper (35) (10,858 bp; D3H54_RS30220 to D3H54_RS30270) and contains genes enabling the catabolism of IB to 2-hydroxyisobutyrate (2-HIBA) (Fig. 3B; Tables S2 and S3). Six genes (*ibcABCDEF*) comprise a predicted Group 2 soluble di-iron monooxygenase (SDIMO), hereafter referred to as isobutylene monooxygenase (IMO), that putatively catalyzes the first reaction in isobutylene catabolism. These genes encode an α-oxygenase subunit (*ibcA;* 58.1 kDa), a *β*-oxygenase subunit (*ibcE*; 38.3 kDa), and a *γ*-oxygenase subunit (*ibcB*; 10.2 kDa), as well as a Riske-type ferredoxin (*ibcC*; 12.5 kDa), an effector/coupling protein (*ibcD*; 12.3 kDa), and a reductase (*ibcF*; 36.3 kDa). An epoxide hydrolase gene (*ibcK*; 34.6 kDa), an alcohol dehydrogenase (*ibcH*; 59.8 kDa), and an aldehyde dehydrogenase (*ibcJ*; 52.1 kDa) are also present in cluster 1. The corresponding enzymes enable the hydrolysis of IBO to MPD (12) and the further oxidation of MPD to 2-HIBA, respectively (15, 16). Alignment comparisons for these gene products with proteins of known function are shown in Table S2. Cluster 1 also contains a hypothetical protein (*ibcI*; 16.0 kDa) of unknown function as well as an additional aldehyde dehydrogenase (*ibcG*; 46.8 kDa), which does not have a predicted role in the epoxide-generating pathway of ELW1. One reasonable possibility is that this aldehyde dehydrogenase participates in the further oxidation of 2-methyl-2-propene-1-ol. This alcohol supports the growth of ELW1 and has been suggested previously to be a minor byproduct of IB catabolism, resulting from the monooxygenase-catalyzed oxidation of IB to an alcohol rather than an epoxide (6).

Cluster 1 is flanked on either side with predicted mobile element genes (Fig. S2). For example, upstream of *ibcA*, there are two predicted recombinase/integrase genes and a helix-turn-helix domain-containing gene on the same strand as well as four IS-like/transposase genes on the opposing strand. Immediately downstream of *ibcK*, there is another predicted transposase gene followed by another predicted monooxygenase, a Group 6 SDIMO, which is structurally distinct from IMO (Fig. S2). The GC content of cluster 1 (58.7% GC) is lower than the rest of the plasmid (65.9% GC), and there are also some large variations in codon usage, particularly cysteine with 55.9% TGC/44.1% TGT in the cluster compared to 80.1% TGC/19.9% TGT in the plasmid and asparagine with 49.5% AAC/50.5% AAT in the cluster compared to 81.7% AAC/18.3% AAT in the plasmid. The data and the insertion sequences flanking *ibc* cluster 1 in pELW1-1 (Fig. S2) all suggest that the predicted operon has been incorporated into the plasmid through transposition and horizontal gene transfer.

### *ibc* cluster 2

Cluster 2 (37,153 bp; D3H54_RS30025 to D3H54_RS30180) has 32 coding sequences across both strands and multiple predicted operons (35) (Fig. 3C; Tables S2 and S3). This cluster contains genes that align with 2-HIBA-CoA ligase (*ibcN*; 52.8 kDa) (16, 17), a cobalamin-dependent methylmalonyl-CoA mutase (*ibcM*; 15.3 kDa and *ibcP*; 64.0 kDa), and a gene that encodes an auxiliary protein (*ibcO*; 34.2 kDa) that may stabilize the dimer form of the mutase and protect the cobalamin cofactor from reactive intermediates and irreversible inactivation (18–20, 36). Genes encoding a 3-hydroxybutyryl-CoA dehydrogenase (*ibcQ*; 30.6 kDa) (37) and acetyl-CoA C-acetyltransferase (*ibcL*; 41.7 kDa) (18, 37) are also present. Other genes within cluster 2 also include a predicted transposase, a FadR/GntR family transcriptional regulator, a branched chain amino acid synthesis gene (ketol-acid reductoisomerase; *ilvC*), an MHS family MFS transporter, a histidine phosphatase family protein, and a thiamine pyrophosphate-dependent lyase (D3H54_RS30180) that may be involved in a minor route of 2-HIBA catabolism (Table S2) (16). Amino acid alignment comparisons for these gene products to proteins with known functions are shown in Table S2.

ELW1 requires Co^2+^ ions to grow on IB and all IB catabolism intermediates preceding 3-hydroxybutyrate (6). The methylmalonyl-CoA mutase likely responsible for converting 2-HIBA-CoA to 3-hydroxybutyryl-CoA also utilizes adenosylcobalamin (B_12_) as a cofactor (14, 18). Notably, multiple genes encoding enzymes involved in cobalamin biosynthesis are adjacent to the mutase-encoding genes, so these genes were included in our definition of *ibc* cluster 2. Using uroporphyrinogen III as the starting precursor, gene annotation revealed a near-complete cobalamin biosynthesis pathway (38) (Fig. S3; Table S3) with the absence of only the predicted gene for *cobG* (precorrin-3B synthase), which is found chromosomally (Fig. S3). A detailed list of features and predicted functions of cluster 1 and 2 genes is shown in Table S3. There is an insertion sequence midway through cluster 2 and a predicted integrase upstream of cluster 2, which suggests the full Ibc pathway may have arisen from separate recombination events (Fig. S2).

### *ibc* cluster comparison with other IB-catabolizing strains

The *ibc* clusters from ELW1 share significant gene organization and nucleotide sequence identities (cluster 1 = 99.7%; cluster 2 = 99.6%) with gene clusters in the recently characterized IB-catabolizing strain, IBE100 (Table S2; Fig. S2 and S4) (5). The major difference between the clusters in these two bacteria is the region of DNA between the end of cluster 2 and the beginning of cluster 1. In ELW1, this region is 7,783 bp and encodes two predicted ATP-binding proteins, two integrases, two transposases, and one helix-turn-helix domain-containing protein. In IBE100, this region is 5,300 bp and encodes two predicted transposases and two integrases. There are more substantial differences between the *ibc* clusters in ELW1 and IBE200 (Fig. S2 and S4). Unlike in ELW1, the genes for *ibcH*, *ibcI*, *ibcJ*, and *ibcK* in IBE200 are not positioned adjacent to the genes encoding IMO (*ibcA–F*) but are directly upstream of the IBE200 cluster 2. In addition, IBE200 has two additional predicted regulatory genes (one helix-turn-helix protein and one AraC family protein) within the *ibc* clusters that have low sequence homology (<40%) to any genes in ELW1. IBE200 has no predicted insertion or transposition sequences within or directly surrounding its *ibc* clusters. Finally, although present, the organization of cobalamin biosynthesis genes in IBE200 compared to ELW1 is slightly different, and there are three additional predicted genes in the IBE200 cluster 2 (a predicted metallopeptidase, a B-4DMT family transporter, and a nitrite reductase).

Interestingly, all three known IB-catabolizing strains possess an aldehyde dehydrogenase (*ibcG*) immediately downstream of the Group 2 SDIMO (*ibcA–F*) (Fig. S4). Other known alkene oxidizers like *Rhodococcus* sp. AD45 and *X. autotrophicus* Py2 also encode predicted aldehyde dehydrogenases immediately downstream of their respective alkene-oxidizing Group 2 SDIMO encoding genes. This strongly suggests an evolutionary retention of this aldehyde dehydrogenase homolog in alkene catabolism.

Despite some interesting similarities, the lack of ungapped, fully contiguous assemblies for IBE100 and IBE200 makes it challenging to determine if the related *ibc* clusters in these strains are plasmid-borne. Like pELW1-1, the large IBE200 contig (175,455 bp) containing similar *ibc* genes to those in strain ELW1 also contains predicted plasmid partitioning genes *parA* and *parB*. However, the *par* genes are not found in the much larger *ibc* gene-containing contig (357,582 bp) of IBE100. Within the large IBE100 contig, there is a predicted gene for *mobF*, which is also present in pELW1-1 and IBE200. This gene is often associated with conjugative plasmid transfer (39). A more complete comparison of pELW1-1 and the two IBE strain contigs that contain *ibc* clusters is shown in Fig. S5. Overall, the differences between pELW1-1 and the IBE100 and IBE200 large contigs that contain comparable *ibc*-related genes suggest that the *ibc* clusters, despite similarities, have different acquisition and evolutionary histories.

### Growth and whole-cell enzymatic assays of ELW1 and ELW1ΔpELW1-1

Strain ELW1ΔpELW1-1 that lacked the large plasmid, pELW1-1, was isolated by serial passage on nutrient-rich agar medium while screening for the lack of growth on IB, as described in Materials and Methods. To investigate the role of this plasmid in IB catabolism, the abilities of strains ELW1 and ELW1ΔpELW1-1 to grow on IB and established intermediates of IB catabolism were compared (Table 1). The wild-type strain ELW1 grew on all the tested substrates. In contrast, strain ELW1ΔpELW1-1 did not grow on any IB-related metabolite (IB, IBO, MPD, or 2-HIBA) but retained the ability to grow on fructose and *n*-octane (Table 1). The latter two substrates can be catabolized by enzymes encoded by genes found only on the chromosome.

**TABLE 1:** Monooxygenase activity of strains after growth on various substrates

Strain	Growth substrate^*a*^	OD_600_^*b*^ (no. of days)	Specific activity^*c*^
ELW1	Fructose	1.39 ± 0.11 (3)	0.5 ± 0.4
	Fructose + IB	1.54 ± 0.08 (3)	10.1 ± 0.2
	IB	1.52 ± 0.13 (5)	15.8 ± 6.2
	IBO	0.85 ± 0.02 (7)	2.9 ± 0.0
	MPD	0.62 ± 0.03 (7)	3.2 ± 0.2
	2-HIBA	0.83 ± 0.06 (7)	Undetected
	*n*-octane	0.54 ± 0.01 (9)	Undetected
ELW1ΔpELW1-1	Fructose	0.89 ± 0.06 (6)	Undetected
	Fructose + IB	1.25 ± 0.10 (5)	Undetected
	IB	NG	NG
	IBO	NG	NG
	MPD	NG	NG
	2-HIBA	NG	NG
	*n*-octane	0.58 ± 0.02 (9)	Undetected

^*a*^ 0.5 mmol of liquid substrate or 10% (vol/vol gas phase) of IB were used in cultures. IB at ~10% (vol/vol) of the headspace is the normal growth condition for ELW1 using this substrate.

^*b*^ Initial culture OD_600_ ≤ 0.02. Data represent the mean and SD with days of growth (in parentheses) of three biological replicates. NG, no growth (monitored for 35 days).

^*c*^ Specific activity units are nmol TBA min^−1^mg total soluble protein^−1^. Data represent the mean and SD of three biological replicates, except for ELW1 grown on fructose alone and IB alone. ELW1 grown on fructose or IB, the data represent the combined means from two separate assays (*n* = 3 and *n* = 2). In all cases, the limit of detection for TBA production was >0.2 nmol TBA min^−1^ mg total protein^−1^.

The specific activity of IMO was investigated following the production of TBA from the oxidation of isobutane (2-methylpropane) (Table 1). Isobutane is a non-growth-supporting IMO substrate, and unlike gaseous alkenes that are oxidized by IMO to epoxides that are further transformable in whole cells, TBA is a terminal and stable reaction product that can be readily detected by gas chromatography (GC) (40). The fastest rate of TBA production (~16 nmol min^−1^ mg protein^−1^) was observed for IB-grown cells of ELW1, while lower rates (~3 nmol min^−1^ mg protein^−1^) were observed for cells grown on IBO or MPD. No activity was detected for ELW1 cells grown on either 2-HIBA or *n*-octane. Cells of ELW1 grown on fructose exhibited low rates of TBA production (~0.5 nmol min^−1^ mg protein^−1^), but this increased ~20-fold when IB was added to the culture. In contrast, no TBA production was observed for strain ELW1ΔpELW1-1 regardless of the growth substrate used. These data implicate the pELW1-1 encoded IMO as the principal monooxygenase that initiates IB catabolism.

### Activity-based labeling

**Fig 4:**
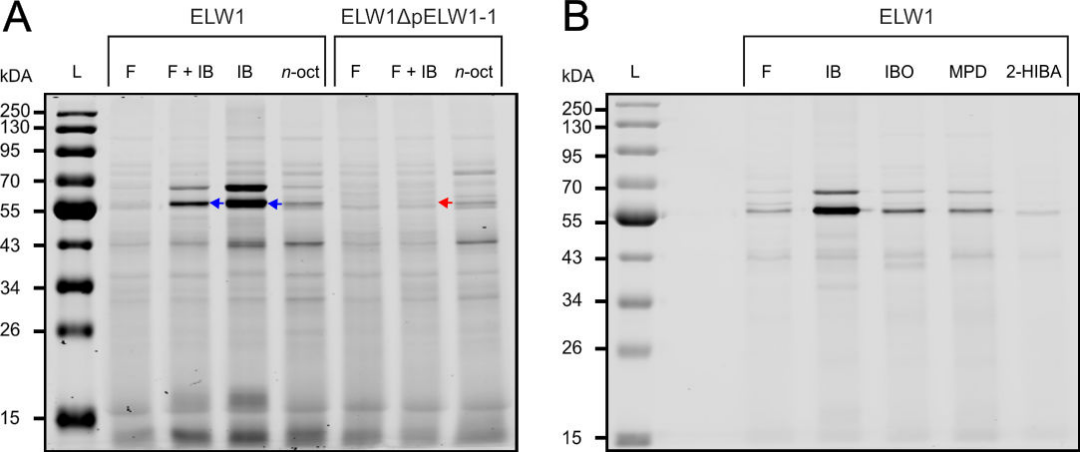
Fluorescent labeling patterns for ELW1 and ELW1ΔpELW1-1 after growth on various substrates. (**A**) L, PageRuler Prestained NIR Protein Ladder; F, fructose; F + IB, fructose plus isobutylene; IB, isobutylene; and *n*-oct, *n*-octane. Blue arrows indicate the putative fluorescently labeled IbcA protein band in strain ELW1. Red arrows indicate the missing fluorescently labeled IbcA protein band in strain ELW1ΔpELW1-1. (**B**) L, PageRuler Prestained NIR Protein Ladder; F, fructose; IB, isobutylene; IBO, isobutylene oxide; MPD, 2-methyl-1,2-propanediol; and 2-HIBA, 2-hydroxyisobutyric acid. Panel A (lanes F and IB) and panel B (lanes F and IB) are from distinct biological cultures.

ABL is a useful tool for detecting and identifying catalytically active enzymes in complex biological mixtures (41). In addition to the whole-cell activity assays described above, ABL was also used to investigate monooxygenase expression levels in strains ELW1 and ELW1ΔpELW1-1 (42–44). Resting cells grown on various substrates were treated *in vivo* with a diyne probe (1,5-hexadiyne [15HD]), reacted *in vitro* with a fluorophore, and subjected to SDS-PAGE and NIR imaging, as described in Materials and Methods. A highly fluorescent ~58 kDa band was observed for cells grown on either IB alone or fructose plus IB (Fig. 4A and B). The apparent mass of this band matches the predicted mass of IbcA, the IMO α-oxygenase subunit. This is the active site-containing component of the putative IB-oxidizing monooxygenase and the expected target of ABL approaches used in this study (45). Lower, but still detectable, levels of fluorescence for the ~58 kDa band were observed for cells of ELW1 grown on either IBO, MPD, fructose, or 2-HIBA (Fig. 4B). The results of these ABL assays, therefore, closely reflect the trends observed for the whole-cell assays of isobutane-dependent TBA production (Table 1). In addition to the 58 kDa fluorescent band, another highly fluorescent ~68 kDa band was observed in ELW1 cells grown either on IB or fructose plus IB (Fig. 4A and B). In contrast, neither the ~58 nor the 68 kDa fluorescent bands were detected for cells of ELW1ΔpELW1-1 grown on fructose or fructose plus IB (Fig. 4A).

### LC-MS/MS identification of fluorescently labeled proteins

The proteins present in the ~58 and 68 kDa fluorescent bands observed for IB-grown cells of ELW1 were determined by excising Coomassie-stained bands at the equivalent migration point as the fluorescent bands. After in-gel tryptic digestion and analysis by LC-MS/MS, 632 and 468 proteins were detected and identified for the ~58 and the ~68 kDa fluorescent bands, respectively. The most abundant protein identified in the ~58 kDa fluorescent band was IbcA (Table S4). Although the putative IMO from ELW1 has not been either purified or heterologously expressed, several lines of evidence point to this being the enzyme responsible for initiating IB catabolism. The gene arrangement of Ibc α-𝛾-F-C-*β*-R indicates this enzyme is a Group 2 SDIMO and further suggests the multimeric oxygenase component of IMO is likely to exist *in vivo* as an (ɑ*β𝛾*)^2^ configuration, as shown for purified Group 2 monooxygenases, including Xamo from *X. autotrophicus* Py2, the isoprene-oxidizing IsoMO from *Rhodococcus* sp. AD45, and toluene-4-monooxygenase from *Pseudomonas mendocina* KR1 (46–48). Further catalytic similarities include the limited sensitivity of all of these enzymes to inactivation by the simplest alkyne, acetylene, while also being irreversibly inactivated by longer chain terminal alkynes, including the diynes that form the foundation of our ABL approach (43, 44). Finally, all of these Group 2 SDIMOs can oxidize a wide range of alkenes, including chlorinated alkenes such as trichloroethylene (49–52), even though the primary physiological function of some of these enzymes is to oxidize aromatic compounds, such as toluene (53, 54). With this last point in mind, it has also been demonstrated that IB-grown cells of ELW1 can readily oxidize the polyaromatic hydrocarbon, phenanthrene (55), while propylene-grown *X. autotrophicus* Py2 and isoprene-grown *Rhodococcus* sp. AD45 have been shown to oxidize simple aromatics (52, 53).

The most abundant protein identified for the ~68 kDa fluorescent band was IbcH, an alcohol dehydrogenase (Table S4). The ABL approach used in this study targets monooxygenases based on their ability to catalytically activate one of the two alkyne groups on ABL probes such as 15HD. Consequently, fluorescent labeling of IbcH using this approach was unexpected. One potential explanation for this observation and the detection of other labeled proteins using ABL may be due to non-specific labeling reactions caused by the release of an activated ABL probe from the active site of IMO and subsequent adventitious binding of the probe to other abundant cellular proteins or specific proteins that are closely located to IMO (44). Non-specific off-target labeling can also occur due to reactive thiotriazoles generated from cysteine-free thiols under a range of copper(I)-catalyzed alkyne–azide cycloaddition (CuAAC) conditions (56). Finally, acetylenic compounds such as 3-butyn-1-ol can inactivate alcohol dehydrogenases (57), and this could underlie the unexpected labeling of IbcH if IMO generates an alcohol derivative of 15HD. Nonetheless, IbcH, as the enzyme predicted to catalyze the third reaction in the putative Ibc pathway, appears as an abundant, labeled protein in the ABL analysis.

### RNA-seq and proteomic analysis of ELW1 during isobutylene catabolism

To further investigate the role of plasmid-encoded genes in IB catabolism, RNA-seq and proteomic analyses were conducted for ELW1 cells grown either on IB or fructose. Out of 6,246 transcripts identified by differential expression analysis, 296 genes were upregulated, and 289 genes were downregulated (|log_2_FC| value ≥ 1) in IB-grown cells. Some of the upregulated chromosomal genes are predicted to serve in functions related to stress responses (universal stress proteins; hypoxia response regulators), nutrient transport and virulence (MFS transporters; Type VII secretion proteins), amino and fatty acid synthesis and catabolism (keto acid reductoisomerase; 3-oxoacyl-ACP synthase; methylmalonate-semialdehyde dehydrogenase), and carbon storage (acetoin reductase; 2,3-butanediol dehydrogenase). These trends are similar to those observed in IB-grown IBE200 cells (5).

**Fig 5:**
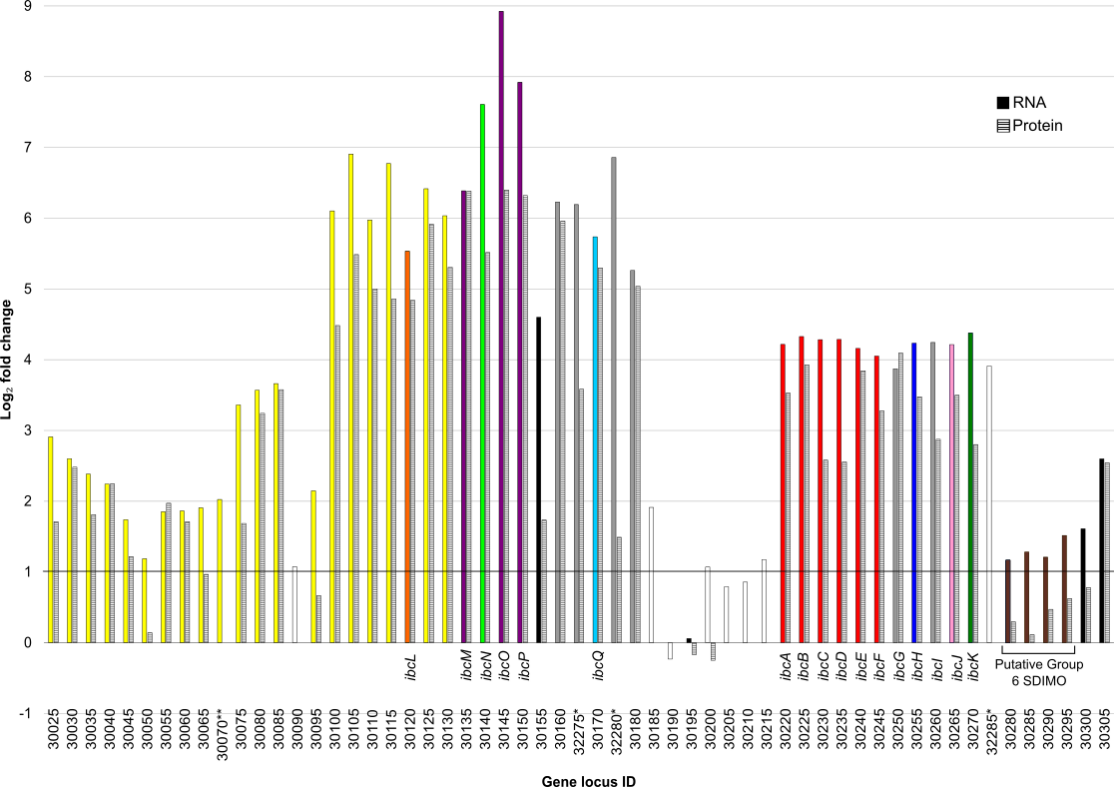
Differential expression levels of *ibc* gene clusters and products. Transcriptomic and proteomic data are from IB-grown versus fructose-grown cells. RNA-seq data are represented by solid-colored bars, with corresponding proteomic data represented by striped black and white bars. Color coding corresponds to Fig. 2 and 3, Tables S2 and S3, and Fig. S2 and S4. Gene loci designations are from accessions NZ_CP032155.1 and NZ_CP032156.1. Data represent the means (RNA, *n* = 5; protein, *n* = 3) from biological replicates. The solid horizontal dark line at log_2_ fold change = 1 represents our arbitrary cutoff of significantly upregulated genes. A positive log_2_ fold change represents induction in the presence of IB. * represents partial transcripts that were not matched definitively with the predicted annotated sequence. ** represents an identified protein that could not be distinguished from the chromosome-encoded homologs. Transcripts at loci at 30190–30200 had adjusted *P*-values (FDR) ≥ 0.05; all other transcripts had adjusted *P*-values (FDR) ≤ 0.05. All proteins identified, except for 30200, had *P*-values ≤ 0.05. Proteins for 30090, 30185, 30190, 30195, 30205, and 30210 were not identified.

Of the upregulated genes, 72 were found on pELW1-1, with the majority being from the two *ibc* gene clusters (Fig. 5; Table S3). The genes with the highest upregulation (log_2_FC > 7) within these clusters were *ibcN*, *ibcO*, and *ibcP*, all of which are involved in the transformation of 2-HIBA to 3-hydroxybutyryl-CoA. All *ibc* cluster 1 genes were upregulated to similar levels (log_2_FC ~ 4.21 ± 0.13). The only genes that were not upregulated within and adjacent to the two *ibc* clusters encode transposases and insertion sequences (D3H54_RS30190, RS30195, RS30205, and RS30210). Transcripts for D3H54_RS32285 were not detected. Of all genes, both chromosomal and plasmid-borne, the *ibc* genes had the highest average upregulation (log_2_FC > 3.9), and there were no significantly downregulated plasmid-borne genes.

The cobalamin biosynthesis genes found in cluster 2 were all upregulated to some degree. Although the plasmid-encoded cobalamin biosynthesis genes have paralogs on the chromosome, none of these chromosomal genes were found to be substantially upregulated (Table S5). *cobG* from the chromosome, the missing gene from the cluster 2 canonical aerobic cobalamin biosynthesis pathway, was also not upregulated. The chromosomal genes for uroporphyrinogen III biosynthesis were also not upregulated, likely indicating that a basal level of expression for these genes is sufficient for cobalamin-dependent growth on IB.

Of 4,718 proteins detected by differential expression analysis, 158 proteins were upregulated, and 229 proteins were downregulated. Most of the gene products from the *ibc* clusters were significantly upregulated except for several predicted cobalamin biosynthesis enzymes (D3H54_RS30050, RS30065, and RS30095) (Table S3). As with the RNA-seq results, none of the chromosome-encoded cobalamin biosynthesis enzyme homologs were found to be significantly upregulated except for CbtA (D3H54_RS26145, log_2_FC = 1.14) (Table S5). The gene product for the IS110 transposase (D3H54_RS30090) was undetected, and the product for plasmid-borne *cbtB* (D3H54_RS30070) could not be differentiated from the chromosome-encoded homologs. The proteins with the highest upregulation (log_2_FC > 6) were again found in cluster 2 and included IbcO, IbcM, and IbcP. All other Ibc proteins were significantly elevated as well, albeit cluster 1 gene products displayed more irregular fold changes compared to the transcriptional data (log_2_FC of 2.5–4.1).

Although there is strong evidence that IMO of ELW1 is responsible for initiating IB catabolism, the genome of this strain encodes several other monooxygenases. Chromosomally, there is a predicted alkane 1-monooxygenase (AlkB), multiple predicted cytochrome P450s, and other predicted monooxygenase family proteins. Although some of these enzymes may be able to oxidize alkenes such as IB (58–60), the alkane 1-monooxygenase (AlkB) is most likely responsible for initiating the growth of ELW1 on *n*-octane. This enzyme was neither transcriptionally (log_2_FC = −0.18) nor proteomically (log_2_FC = −0.25) upregulated during growth on IB. Two chromosomally encoded cytochrome P450s and a NAD(P)/FAD-dependent oxidoreductase were significantly upregulated in IB-grown cells. However, the inability of the plasmid-free strain ELW1ΔpELW1-1 to grow on IB, oxidize isobutane to TBA (Table 1), and express ABL-detectable proteins (Fig. 4) indicates that none of these chromosomally encoded enzymes participate directly in IB catabolism.

Another monooxygenase potentially involved in IB catabolism is the Group 6 SDIMO encoded downstream of cluster 1. However, no significant upregulation of protein was observed for this multicomponent enzyme in IB-grown ELW1 cells compared to its corresponding transcriptional upregulation (Fig. 5; Table S3). Furthermore, differential expression fold changes were highest beginning with the predicted Group 6 SDIMO-associated sensor kinase (D3H54_RS30305) but then diminished with the remaining proteins (Table S3). There are homologies and shared gene expression patterns between the *ibc* gene clusters in ELW1, IBE100, and IBE200, including the adjacent genes for the Group 6 SDIMO (Fig. S2). For example, the Group 6 SDIMO was not significantly transcriptionally upregulated in IB-grown IBE200 and was not identified by peptide-mass fingerprinting in IB-grown IBE100 cells (5). Finally, there is no evidence that ELW1 can grow on short-chain alkanes (C2–C6) that are known to be oxidized by Group 6 SDIMOs (6). Collectively, these observations suggest that the Group 6 SDIMO genes that are consistently found in IB-metabolizing bacteria to date are not directly involved in the catabolism of IB.

Currently, the regulation of IB catabolism in ELW1 is not understood. The reads from the RNA-seq data presented in this study (see Materials and Methods) suggest that cluster 1 is transcribed from a common start site and would comprise a single operon. A promoter and operon prediction algorithm (35) also places a promoter upstream in *ibcA*, the first IMO gene in cluster 1 (data not shown). Overall, genes in cluster 1 are upregulated in unison upon exposure to IB, although no obvious regulatory genes are present in or adjacent to this cluster. It is tempting to speculate that the Group 6 SDIMO genes and, importantly, the adjacent DNA-binding domain-encoding genes have been retained for cluster 1 *ibc* gene regulation rather than for a direct catalytic role in IB catabolism. These observations warrant further investigation into *ibc* gene regulation.

### Conclusion

In this study, we report the complete annotated genome of *Mycolicibacterium* sp. ELW1 and identify two gene clusters associated with IB catabolism that are present on a single-copy megaplasmid, pELW1-1. The loss of this plasmid resulted in the inability of ELW1 to grow on IB and the immediate downstream intermediates of IB catabolism but had no effect on the growth on other substrates. Concurrent RNA-seq and proteomic expression analyses demonstrate that the genes within both clusters are strongly upregulated by IB. The importance of the cluster gene products, with a focus on the monooxygenase (IMO) that putatively initiates IB catabolism, was confirmed using whole-cell monooxygenase assays and ABL of the *α*-oxygenase subunit, IbcA. These results establish that pELW1-1 is required for IB catabolism by strain ELW1 and that two plasmid-borne IB-inducible gene clusters encode the Ibc pathway.

## MATERIALS AND METHODS

### Reagents

The organic chemicals used in this study, their purities, and the suppliers are as follows: Alexa Fluor 647 azide, triethylammonium salt (99% purity; Invitrogen, Grand Island, NY, USA); Tris-hydroxypropyltriazolylmethylamine (95% purity; Lumiprobe, Hunt Valley, MD, USA); 1,5-hexadiyne (50% [vol/vol] in pentane; Alfa Aesar, Ward Hill, MA, USA); 2-methyl-1,2-epoxypropane (isobutylene oxide, 99+%; Alfa Aesar, Ward Hill, MA, USA); 2-methyl-1,2-propanediol (Oakwood Chemical, Estill, SC, USA); PageRuler Prestained NIR Protein Ladder (Thermo Scientific, Waltham, MA, USA). 2-methylpropene (isobutylene, 99% purity), *α*-hydroxyisobutyric acid (2-HIBA; 98% purity), *n*-octane (>99% purity), *tertiary*-butyl alcohol ( 99.5% purity), and fructose (>99% purity) were obtained from Sigma-Aldrich Chemical Co. (Milwaukee, WI, USA). 2-methylpropane (isobutane; CP grade) and compressed gases (H_2_, N_2_, and air) were obtained from local industrial vendors (Airgas, Raleigh, NC, USA).

### Bacterial cultivation and media

*Mycolicibacterium* sp. strain ELW1 was maintained on mineral salt medium (MSM) agar (1.5%) plates in a glass desiccator containing IB (∼10%, vol/vol, gas phase) and passaged every 3–4 weeks for the duration of this study (6). *Mycolicibacterium* sp. strain ELW1ΔpELW1-1 was maintained on plant count agar (PCA) that contained casein-yeast extract-dextrose medium (Becton, Dickinson and Co., Sparks, MD, USA) and was passaged every 3–4 weeks for the duration of this study.

Cultures of ELW1 were routinely grown on IB in MSM media (100 mL) in previously autoclaved glass serum bottles (700 mL) (Wheaton Scientific, Millville, NJ, USA) sealed with sterile open-top caps fitted with butyl rubber septa, as described previously (6). Unless otherwise stated, the cultures were inoculated to an initial OD_600_ ≤ 0.01–0.05 with a suspension of cells previously grown on MSM and IB (10%, vol/vol, gas phase). IB (~10%, vol/vol, gas phase) was added to the sealed bottle using sterile plastic syringes fitted with a disposable 0.1 μm filter (Millipore Co., Bedford, MA, USA). The bottles were incubated at 30°C in the dark using a shaker operated at 150 rpm. Growth was measured as optical density at 600 nm (OD_600_) with a spectrophotometer (WPA CO8000 Cell Density Meter; Biochrom, Cambridge, UK). To confirm the purity of cultures after growth, a culture sample (25 µL) was streaked onto non-selective PCA plates.

Batch cultures were also grown on fructose, *n-*octane, IBO, MPD, 2-HIBA, or a combination of these substrates, as described above. For fructose, IBO, MPD, 2-HIBA, and *n*-octane-grown cultures, 0.5 or 1 mmol of substrate (fructose and 2-HIBA: 1 M filter-sterilized aqueous solution; *n*-octane, IBO, and MPD: filter-sterilized neat compound) was added to the culture medium. In the case of the plasmid-free strain ELW1ΔpELW1-1, growth was monitored for up to 35 days. For ABL and whole-cell activity assays, cells were grown as described above and then harvested at late log–stationary phase from the culture medium, washed, and then finally resuspended in phosphate buffer (PB; 50 mM NaH_2_PO_4_, pH 7.0), as described previously (44).

### Curing of the megaplasmid pELW1-1

Plasmid curing of strain ELW1, to ELW1ΔpELW1-1, was conducted under non-selective conditions by continuous passage. Frozen stocks of ELW1 were initially grown on MSM agar with IB (∼10%, vol/vol, gas phase), as described above. After 3 days, 50 colonies were patched by replica plating onto a new MSM plate and a PCA plate. As described before, the MSM plate was supplied with IB (∼10%, vol/vol, gas phase). After an additional 3 days, patches were examined and struck from the PCA plate onto both a new MSM and PCA plate. After subsequent passages (≥10), patches that demonstrated loss of growth on the MSM plate were cultured on a larger scale (both on solid and in liquid plate count media) for further analysis. This process was repeated until colonies showed a lack of growth after 3 weeks of incubation in liquid MSM with IB (10%, vol/vol, gas phase). Plasmid loss was subsequently verified by PCR targeting three positions on pELW1-1 (data not shown; primers used are shown in Table S6) and subsequent full genome sequencing detailed below. ELW1ΔpELW1-1 was subsequently maintained on PCA.

### Genome sequencing, assembly, and analysis

#### Initial sequencing

Initial genome sequencing of ELW1 was done in 2019 by extracting DNA using the UltraClean Microbial DNA Isolation Kit (Mo Bio Laboratories 1224-50, Carlsbad, CA, USA) following the manufacturer’s protocol except for cell disruption, which used a FastPrep-24 MP Bio homogenizer (Solon, OH) at 4 m/s for 20 s in a 2 mL tube containing 0.1 mm silica beads (MP Biomedicals #6911-100, Santa Ana, CA, USA). Genomes were obtained using PacBio sequencing technology (RTL Genomics, Lubbock, TX, USA). A DNA library was prepared using SMRTbell Template Prep Kit 1.0 (PacBio 100-259-100, Menlo Park, CA, USA) with an average final size of 18.9 kb. Cells were sequenced on two PacBio 10 kb cells. CLC Genomics Workbench, version 9.5 (QIAGEN, Aarhus, Denmark), with the Genome Finishing Module, was used for assembly with the PacBio *De Novo* Assembly Pipeline. The resulting chromosome contig was reordered in CLC Genomics Workbench to reposition the start site at 100 bases upstream of the start codon of DnaA. Initial annotation was accomplished with the Rapid Annotation using Subsystems Technology (61). Annotation was also accomplished with the GenBank pipeline. Further analysis in clusters of genes of interest was individually submitted to BLAST, and the annotation of the gene and/or protein was confirmed. Genomic sequences were submitted to GenBank under the BioProject PRJNA489466, BioSample SAMN09980424, and accessions CP032155.1 (genome) and CP032156.1 (pELW1-1).

#### Resequencing

Strains ELW1 and ELW1ΔpELW1-1 were sequenced in 2024 for the analysis of plasmid loss using Oxford Nanopore Technology (Oxford, UK) services of Plasmidsaurus (Eugene, OR, USA). Cell pellets were grown as described previously and suspended in Zymo 1× DNA/RNA Shield (Irvine, CA, USA) per the manufacturer’s protocol. DNA extraction and bacterial genome sequencing were performed by Plasmidsaurus using Oxford Nanopore Technology (ONT) (Hybrid ONT + Illumina) with custom analysis and annotation. Genome assembly statistics are shown in Table S1. Genomic sequences were submitted to GenBank under the BioProject PRJNA1269221, BioSample SAMN48782278, and accessions CP193805.1 (genome) and CP193804.1 (pELW1-1). Genomic sequences for ELW1ΔpELW1-1 were submitted to GenBank under the BioProject PRJNA1269221, BioSample SAMN48836137, and accession CP194511.1 (genome).

#### Genome analysis

Genome sequences were aligned and compared using CLC Genomics Workbench version 23.0.4 (QIAGEN) Whole Genome Alignment plugin, including the Create Whole Genome Alignment tool with default settings. Determination of average nucleotide identity and alignment coverage percentage used the Create Average Nucleotide Identity Comparison function with default settings, using the full genome or chromosome only unless otherwise stated. Analysis of areas of interest and specific genes was submitted to BLAST and/or examined with SnapGene version 8.0.3 (https://www.snapgene.com/). Unless otherwise specified, potential operons were identified with *Operon-mapper* (35). All figures and analyses were created using NCBI ELW1 RefSeq sequences NZ_CP032155.1 and NZ_CP032156.1. All figures were created using SnapGene software (version 8.0.3) (https://www.snapgene.com/), CLC Genomics Workbench version 23.0.4 (QIAGEN), and Inkscape Project 2020 (https://inkscape.org).

### Activity-based labeling and whole-cell activity assays

For both ABL and GC-based whole-cell assays of IMO abundance and activity, batch cultures of ELW1 and ELW1ΔpELW1-1 were grown with fructose (0.5 mmol), IB (10%, vol/vol, gas phase), fructose (0.5 mmol) supplemented with IB (10%, vol/vol, gas phase), IBO (0.5 mmol), MPD (0.5 mmol), 2-HIBA (0.5 mmol), or *n*-octane (0.5 mmol) as sole carbon and energy sources, as described in Materials and Methods. After 3–9 days, cells were harvested by centrifugation (10,000 RCF for 5 min at 4°C), washed three times in PB (10 mL), and then finally resuspended in PB to an OD_600_of ~4.0.

For ABL analyses, harvested and washed cells (1 mL) were added to a sealed serum vial (10 mL), and 15HD (~1 µmol) was added from a 1 M stock solution in dimethylsulfoxide. The vials were then incubated in a shaking water bath (150 rpm) for 1 h at 30°C. After incubation, cells were harvested by centrifugation (10,000 RCF for 3 min at RT), washed three times with PB (1 mL), and then finally resuspended in PB (250 µL). Lysis matrix B (MP Biomedicals; Irvine, CA, USA) beads (~0.075–0.15 g) were then added to the cell suspension, and the cells were broken using a bead beater using three sequential cycles of 120 s at 5 m/s. The resulting lysate was then centrifuged at 17,000 RCF for 10 min at 4°C to remove unbroken cells and cellular debris. The resulting supernatant was retained, and the protein concentration was determined using the BCA protein assay. A sample (25 µg protein) was then treated with Alexa Fluor 647 azide using a CuAAC (click reaction), as described previously (44). Subsequently, 5 µg of total protein per sample was separated by SDS-PAGE (10% acrylamide gel), and immediately after electrophoresis, AlexaFluor-labeled proteins were detected at 700 nm using a LICOR Odyssey CLx System. The Alexa Fluor 647 azide is ideally excited at 650 nm and has an emission at 671 nm. The LICOR Odyssey CLx System provides two detection channels (700 and 800 nm), with light excitation at 685 and 785 nm, respectively. The gels were then stained with Coomassie Brilliant Blue R-250, destained, and visualized again at 800 nm. In both cases, the resulting images were analyzed with Image Studio Software version 5.2.

The specific activity of IMO in whole cells was determined by quantifying the oxidation of isobutane (2-methylpropane) to TBA (38). Cell suspensions (1 mL) in glass serum vials (10 mL) were sealed with butyl rubber stoppers and aluminum crimp seals. Isobutane (~10%, vol/vol, gas phase) was injected into the vial as an overpressure to initiate the reaction. At *t* = 0 and *t* = 60 min, samples (2 μL) of the reaction aqueous phase were injected into a Shimadzu GC-8A gas chromatograph fitted with a flame ionization detector and a stainless-steel column (0.3 by 183 cm) filled with Porapak Q (80/100 mesh; Waters Associates, Framingham, MA, USA). The instrument was operated with an injector and detector port temperature of 220°C and a column temperature of 180°C. Nitrogen was used as the carrier gas at a flow rate of 15 mL/min.

The GC was interfaced to a Hewlett-Packard HP3395 integrator (Palo Alto, CA, USA) for data collection, and TBA was quantified with a calibration plot generated by adding known amounts of TBA to a reaction vial that contained PB (1 mL) but not cells, as described previously (TBA detection limit > 0.025 nmol) (6, 40). Calibration plots contain at least nine different TBA concentrations, including at least one concentration above the maximal concentration detected in our experiments. All calibration plots were computer fitted by linear regression and had final *R*^2^ values of ≥0.99.

### LC-MS/MS identification of fluorescently labeled proteins

A batch culture of ELW1 was grown on IB (10%, vol/vol, gas phase) until the late log phase, as described above. The culture was then split into two aliquots. One aliquot was used for ABL analyses, as described above, while the other aliquot was untreated. Samples (5 µg total protein) from both aliquots were analyzed by SDS-PAGE using 10% Mini-PROTEAN TGX Precast Gels (Bio-Rad, USA). After SDS-PAGE, the gels were first visualized by fluorescent NIR and then stained with Coomassie Brilliant Blue R-250. The untreated sample was used to determine if ABL had any significant effects on protein banding patterns observed in IB-grown cells of ELW1. The Coomassie-stained gel was submitted to the UNC Metabolomics and Proteomics Core, where they excised the Coomassie-stained bands at the equivalent migration point of the observed fluorescently labeled bands. This was followed by in-gel tryptic digestion, LC-MS/MS analysis, and subsequent protein identification. The LC-MS/MS analysis used a Thermo UltiMate 3000-Exploris 480 and a 90-min Data Dependent Acquisition method. Data were searched in Proteome Discoverer (Thermo Scientific; version 3.1) against the *Mycobacterium* sp. ELW1 reference proteome from Uniprot (https://www.uniprot.org/proteomes/UP000324606) (5,850 sequences) and a common contaminants database (245 sequences). Protein results were filtered for a 1% FDR on the protein and peptide levels, with a minimum of two peptides identified.

### RNA-seq and data-independent acquisition LC-MS/MS proteomic profiling

Batch cultures of strain ELW1 were grown in MSM (100 mL) with IB (10%, vol/vol, gas phase) or fructose (1.0 mmol) to mid-log phase, as described above. The cells were harvested by centrifugation, as previously described (44), split into two aliquots (~3.75 × 10^8^ cells per sample) for either RNA-seq or proteomic profiling, and then pelleted by centrifugation (10,000 RCF for 3 min at RT). The cell pellets were immediately flash frozen in liquid nitrogen and stored at −80°C until used. Cell pellets were then submitted to the North Carolina State Genomic Sciences Laboratory (Raleigh, NC, USA) for RNA extraction, ribosomal depletion, and Illumina RNA library construction and sequencing. Next-generation sequencing was performed using an Illumina NovaSeq 6000 using Block 150 PE 40M PE Reads (Illumina, USA). The raw Illumina reads were de-multiplexed by BCL2FASTQ into FASTQ files for data submission. Raw data can be found at NCBI’s Sequence Read Archive (BioProject: PRJNA1269221, BioSample: SAMN48782278, and accessions: SRR34146425–SRR34146433).

CLC Genomics Workbench version 23.0.4 (QIAGEN) was used to import and process the raw reads, and the RNA-Seq and Small RNA Analysis modules (RNA-seq Analysis and Differential Expression in Two Groups) were used to map the reads to the ELW1 chromosome and plasmid (NZ_CP032155.1 and NZ_CP032156.1) and perform differential expression analysis, respectively, between the two growth conditions (sample sizes: IB-grown, *n* = 4; fructose-grown, *n* = 5). Only genes satisfying the following criteria: |log_2_FC| value ≥ 1 and adjusted *P*-value (FDR) ≤ 0.05 were deemed to meet the selection criteria and significant for further analysis. Sequencing and mapping statistics for RNA-seq are shown in Table S7. A detailed protocol for RNA-seq raw data processing and analysis is included in Table S8.

Cell samples for proteome analysis (sample sizes: IB-grown: *n* = 3 and fructose-grown: *n* = 3) were submitted to the UC Davis Proteomics Core Facility for protein extraction, digestion, and LC-MS/MS analysis using a timsTOF HT/EvoSep. Mass spectrometry data were analyzed by the UC Davis Proteomics Core Facility using Spectronaut version 18.6.231227.55695 (Biognosys Schlieren, Switzerland) using the directDIA workflow with the default settings. Output format for differential expression analysis could not be sorted by chromosome or plasmid, so the investigation of regulated proteins focused mainly on those encoded within the *ibc* clusters and genes of interest on the chromosome. The output format used protein IDs for both CP032155.1 and CP032156.1 and the NZ_CP032155.1 and NZ_CP032156.1 GenBank entries for protein identification. Proteins of interest were identified by GenBank protein ID or name and then matched to the appropriate RNA-seq locus tag.

## Data Availability

Raw data from LC-MS/MS proteomic profiling are available at MassIVE with an FTP download link at ftp://massive-ftp.ucsd.edu/v11/MSV000100012/, while the results of the differential expression analysis from the UC Davis Proteomics Core Facility can be found through https://doi.org/10.5281/zenodo.16985746. This also includes peptide spectrum data as well as the differential expression analysis for the RNA-seq performed in this study. The results from LC-MS/MS identification of fluorescently labeled proteins can be found at https://doi.org/10.5281/zenodo.17780844. RNA-seq and genomic sequencing data are available as described in Materials and Methods.

## SUPPLEMENTAL MATERIAL

Supplemental material aem.02471-25-s0001.pdf Tables S1 to S8; Fig. S1 to S5.

ASM does not own the copyrights to Supplemental Material that may be linked to, or accessed through, an article. The authors have granted ASM a non-exclusive, world-wide license to publish the Supplemental Material files. Please contact the corresponding author directly for reuse.
