# Supplementary material for: Aerobic catabolism of isobutylene by Mycolicibacterium sp. ELW1 requires inducible, plasmid-borne ibc genes
Source: Appl Environ Microbiol. 2026 Apr 17;92(5):e02471-25. doi: 10.1128/aem.02471-25 (PMC13188929; doi:10.1128/aem.02471-25)
Supplement: Supplemental material — Tables S1 to S8; Fig. S1 to S5. [file aem.02471-25-s0001.pdf]

SUPPLEMENTAL MATERIAL

Aerobic catabolism of isobutylene by *Mycolicibacterium* sp. ELW1 requires inducible, plasmid-borne *ibc* genes

John B. Joyce[1], Nicholas W. Faulkner[1], Michael R. Hyman[1,#] and Eric S. Miller[1,#]

[1]Department of Plant and Microbial Biology, North Carolina State University, Raleigh, North Carolina 27695-7615, USA

[#]Corresponding authors: eric_miller@ncsu.edu; michael_hyman@ncsu.edu

Table S1. *Mycolicibacterium* sp. strain ELW1 genome assembly statistics.

Strain Name	Genome size	# of contigs	Genome coverage	CDS	tRNAs/rRNAs	CheckM analysis
ELW1 (2019)	Chromosome: 6,245,979 Plasmid: 221,756	2	86.0x	5,984<br>222	47/6	95.98%
ELW1 (2024)	Chromosome: 6,240,760 Plasmid: 221,734	2	52.0x	5,954<br>223	47/6	100.00%
ELW1ΔpELW1-1	Chromosome: 6,241,844	1	101.0x	5,948	48/6	100.00%

A: ELW1 Whole genome alignments

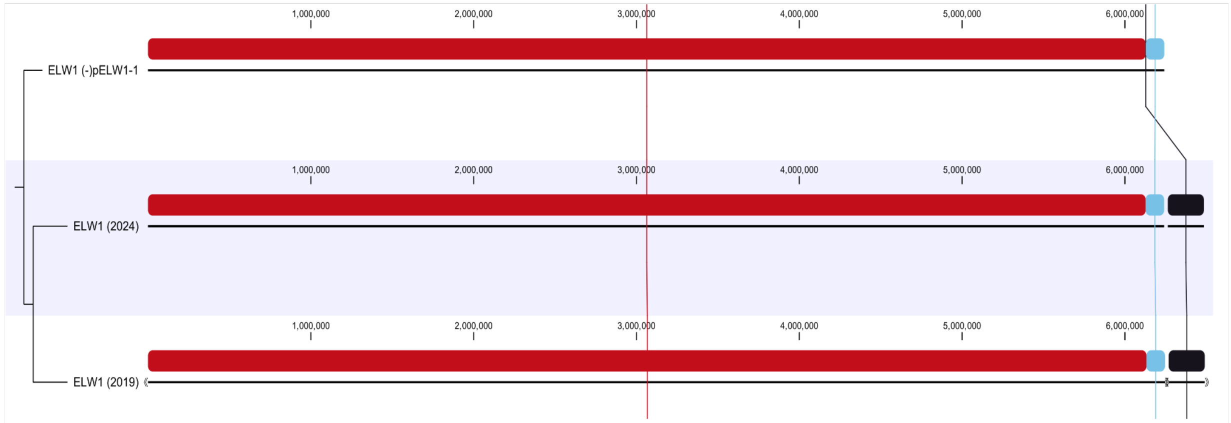

B: Whole genome ANI comparison

		1	2	3
ELW1 (-)pELW1-1	1		100.00	100.00
ELW1 (2019)	2	98.25		100.00
ELW1 (2024)	3	98.29	99.96	

C: Chromosome only ANI comparison

		1	2	3
ELW1 (2019) Chromosome	1		100.00	100.00
ELW1 (-)pELW1-1	2	99.95		100.00
ELW1 (2024) Chromosome	3	99.96	99.99	

Figure S1. Three ELW1 genome sequence alignments.

Genome alignment and average nucleotide identity (ANI) of ELW1 (2019), ELW1 (2024), and ELW1ΔpELW1-1 (designated as ELW1 (-)pELW1-1 in figure). **A**) The taxonomic and genomic alignment comparison between the 2019 ELW1 sequencing and 2024 sequencing results for ELW1 and ELW1ΔpELW1-1. **B**) ANI between the full genomes of ELW1ΔpELW1-1, ELW1 (2019), and ELW1 (2024). **C**) ANI between the chromosomes only for ELW1ΔpELW1-1, ELW1 (2019), and ELW1 (2024). The top diagonal represents the average nucleotide identity comparison between the genomes. The bottom diagonal represents the alignment coverage percentage between the genomes. The results show that the plasmid pELW1-1 is not present in ELW1ΔpELW1-1, while the genomes are essentially identical. Both “2024” genomes were ordered using SnapGene software v8.0.3 (www.snapgene.com) with the +1 base set 100 bp upstream of *dnaA*. The “2024” plasmid pELW1-1 was arranged with +1 100 bp upstream of *parA*. The genome is represented by alignment blocks based on merged seed matches. The plasmid is represented by the small black detached block to the right in both ELW1 (2019) and ELW1 (2024). Alignments were performed using CLC Genomics Workbench v23.0.4 (QIAGEN) Whole Genome Alignment plugin, including the Create Whole Genome Alignment tool with default settings.

Table S2. Comparison of ELW1 IBC pathway enzymes to known homologs.

	ELW1 protein	Homolog/gene locus ID	Species	Total Score	Query Cover (%)	E value	Percent Identity (%)
	IbcA	AmoA<br>IsoA<br>MNO81_04000<br>GKP29_RS08605	*Xanthobacter* sp. strain Py2<br>*Rhodococcus* sp. strain AD45<br>*M. gadium* IBE100<br>*M. paragordonae* IBE200	717<br>862<br>1055<br>901	97<br>97<br>100<br>99	0.0<br>0.0<br>0.0<br>0.0	66.74<br>82.69<br>99.80<br>85.52
	IbcB	AmoB<br>IsoB<br>MNO81_04005<br>GKP29_RS08610	*Xanthobacter* sp. strain Py2<br>*Rhodococcus* sp. strain AD45<br>*M. gadium* IBE100<br>*M. paragordonae* IBE200	113<br>88.2<br>187<br>152	95<br>95<br>100<br>100	2e-39<br>1e-29<br>1e-68<br>8e-55	54.55<br>48.31<br>100.00<br>77.42
	IbcC	AmoC<br>IsoC<br>MNO81_04010<br>GKP29_RS08615	*Xanthobacter* sp. strain Py2<br>*Rhodococcus* sp. strain AD45<br>*M. gadium* IBE100<br>*M. paragordonae* IBE200	115<br>171<br>230<br>151	90<br>99<br>100<br>82	3e-39<br>2e-61<br>7e-85<br>2e-53	52.88<br>65.49<br>99.12<br>75.27
	IbcD	AmoD<br>IsoD<br>MNO81_04015<br>GKP29_RS08620	*Xanthobacter* sp. strain Py2<br>*Rhodococcus* sp. strain AD45<br>*M. gadium* IBE100<br>*M. paragordonae* IBE200	113<br>166<br>223<br>170	94<br>99<br>100<br>94	5e-39<br>2e-59<br>2e-82<br>2e-61	55.34<br>70.37<br>100.00<br>76.70
	IbcE	AmoE<br>IsoE<br>MNO81_04020<br>GKP29_RS08625	*Xanthobacter* sp. strain Py2<br>*Rhodococcus* sp. strain AD45<br>*M. gadium* IBE100<br>*M. paragordonae* IBE200	302<br>458<br>554<br>529	96<br>98<br>81<br>98	8e-106<br>3e-167<br>0.0<br>0.0	48.95<br>67.66<br>100.00<br>75.96
	IbcF	AmoF<br>IsoF<br>MNO81_04025<br>GKP29_RS08630	*Xanthobacter* sp. strain Py2<br>*Rhodococcus* sp. strain AD45<br>*M. gadium* IBE100<br>*M. paragordonae* IBE200	217<br>393<br>687<br>443	96<br>100<br>100<br>100	2e-72<br>2e-141<br>0.0<br>2e-161	39.26<br>56.98<br>99.12<br>66.57
	IbcG	Xaut_4863<br>SZ00_RS00240<br>MpdC?<br>MNO81_04045<br>GKP29_RS08430	*Xanthobacter* sp. strain Py2<br>*Rhodococcus* sp. strain AD45<br>*M. austroafricanum*<br>*M. gadium* IBE100<br>*M. paragordonae* IBE200	138<br>395<br>67.4<br>879<br>517	77<br>98<br>58<br>100<br>100	4e-41<br>2e-139<br>2e-16<br>0.0<br>0.0	32.68<br>52.15<br>26.28<br>99.55<br>60.90
	IbcH	MpdB<br>MNO81_04035<br>GKP29_RS08420	*M. austroafricanum*<br>*M. gadium* IBE100<br>*M. paragordonae* IBE200	368<br>1123<br>1024	96<br>100<br>100	6e-126<br>0.0<br>0.0	41.04<br>99.45<br>92.11
	IbcI	MNO81_04000<br>GKP29_RS08605	*M. gadium* IBE100<br>*M. paragordonae* IBE200	301<br>257	100<br>97	1e-111<br>4e-94	99.34<br>82.88
	IbcJ	MpdC<br>MNO81_04000<br>GKP29_RS08605	*M. austroafricanum*<br>*M. gadium* IBE100<br>*M. paragordonae* IBE200	689<br>980<br>855	100<br>100<br>100	0.0<br>0.0<br>0.0	70.78<br>99.79<br>87.63
	IbcK	EphA<br>MNO81_04050<br>GKP29_RS08435	*A.radiobacter* AD1<br>*M. gadium* IBE100<br>*M. paragordonae* IBE200	431<br>629<br>558	96<br>100<br>96	8e-158<br>0.0<br>0.0	70.41<br>100.00<br>89.00

	ELW1 protein	Homolog/gene locus ID	Species	Total Score	Query Cover (%)	E value	Percent Identity (%)
	IbcL	RePhaA<br>MW290_RS19595<br>MW290_RS04390<br>MNO81_03915<br>GKP29_RS08530	*R. eutropha*<br>*A. tertiaricarbonis* RN12<br>*A. tertiaricarbonis* RN12<br>*M. gadium* IBE100<br>*M. paragordonae* IBE200	329<br>352<br>327<br>805<br>690	100<br>99<br>99<br>100<br>100	1e-114<br>7e-124<br>3e-114<br>0.0<br>0.0	49.13<br>48.13<br>49.25<br>98.76<br>84.79
	IbcM	HcmB<br>MNO81_03930<br>GKP29_RS08555	*A. tertiaricarbonis L108*<br>*M. gadium* IBE100<br>*M. paragordonae* IBE200	155<br>283<br>256	86<br>99<br>98	5e-54<br>8e-105<br>7e-94	60.98<br>100.00<br>90.00
	IbcN	HCL<br>MNO81_03935<br>GKP29_RS08560	*A. tertiaricarbonis L108*<br>*M. gadium* IBE100<br>*M. paragordonae* IBE200	549<br>957<br>847	100<br>100<br>99	0.0<br>0.0<br>0.0	55.70<br>99.57<br>86.27
	IbcO	MeaH<br>MeaB<br>MNO81_03940<br>GKP29_RS08565	*A. tertiaricarbonis L108*<br>*A. tertiaricarbonis RN12*<br>*M. gadium* IBE100<br>*M. paragordonae* IBE200	265<br>158<br>628<br>507	97<br>82<br>100<br>98	3e-91<br>1e-49<br>0.0<br>0.0	48.58<br>35.69<br>99.69<br>84.59
	IbcP	HcmA<br>MNO81_03945<br>GKP29_RS08570	*A. tertiaricarbonis L108*<br>*M. gadium* IBE100<br>*M. paragordonae* IBE200	735<br>1186<br>1069	96<br>100<br>100	0.0<br>0.0<br>0.0	63.29<br>99.83<br>90.97
	IbcQ	FadB<br>Dehydrogenase<br>Dehydrogenase<br>MNO81_03965<br>GKP29_RS08570	*M. tuberculosis*<br>*N. maritimus*<br>*I. hospitalis*<br>*M. gadium* IBE100<br>*M. paragordonae* IBE200	329<br>148<br>158<br>579<br>482	97<br>97<br>96<br>100<br>100	2e-112<br>2e-43<br>2e-45<br>0.0<br>2e-178	56.94<br>31.93<br>36.11<br>99.65<br>82.35
	TPP Lyase (RS30180) (WP_1682 15132.1)	WP_018331913.1<br>ALE72461.1<br>WP_095888858<br>MNO81_03975<br>GKP29_RS08595	*A.chiangmaiensis* DSM 45062<br>*Pseudonocardia* sp. EC080625<br>*Rhodococcus* sp. ACPA4<br>*M. gadium* IBE100<br>*M. paragordonae* IBE200	462<br>482<br>475<br>1138<br>972	98<br>98<br>98<br>100<br>99	6e-161<br>6e-169<br>4e-166<br>0.0<br>0.0	45.55<br>49.47<br>49.20<br>100.00<br>86.47

Amino acid sequences were compared using NCBI Protein BLAST (1). Color coding corresponds to gene clustering in Figure S2 and functional roles. Red text indicates not meeting a typical homology threshold of >30%.

Table S3. Expression levels of *ibc* cluster and adjacent genes during isobutylene catabolism.

	Locus tag ID	Gene Name	NCBI RefSeq Gene Annotation	Predicted Function	log_2 FC RNA	log_2 FC Protein
	D3H54_RS 30025	*cobN*	cobaltochelatase subunit	Cobalamin biosynthesis	2.91	1.71
	D3H54_RS 30030	*cobF*	precorrin-6A synthase (deacetylating)	Cobalamin biosynthesis	2.60	2.48
	D3H54_RS 30035	*cobQ*	cobyric acid synthase	Cobalamin biosynthesis	2.38	1.81
	D3H54_RS 30040	*cobK*	cobalt-precorrin-6A reductase	Cobalamin biosynthesis	2.24	2.25
	D3H54_RS 30045	*cobH*	cobalamin biosynthesis protein	Cobalamin biosynthesis	1.74	1.22
	D3H54_RS 30050	*cobU*	bifunctional adenosylcobinamide kinase/Adenosylcobinamide - phosphate guanylyltransferase	Cobalamin biosynthesis	1.19	0.14
	D3H54_RS 30055	*cobS*	adenosylcobinamide-GDP ribazoletransferase	Cobalamin biosynthesis	1.85	1.97
	D3H54_RS 30060	*cobT*	nicotinate-nucleotide--dimethylbenzimidazole phosphoribosyltransferase	Cobalamin biosynthesis	1.86	1.71
	D3H54_RS 30065	*cbtA*	CbtA family protein	Cobalt transporter	1.91	0.97
	D3H54_RS 30070	*cbtB*	CbtB domain-containing protein	Cobalt transporter	2.03	*
	D3H54_RS 30075	*cobB*	cobyrinate a,c-diamide synthase	Cobalamin biosynthesis	3.36	1.69

	Locus tag ID	Gene Name	NCBI RefSeq Gene Annotation	Predicted Function	log_2 FC RNA	log_2 FC Protein
	D3H54_RS 30080	*cobO*	cob(I)yrinic acid a,c-diamide adenosyltransferase	Cobalamin biosynthesis	3.57	3.24
	D3H54_RS 30085		magnesium chelatase subunit D family protein	Likely cobalamin biosynthesis, potentially gene related to (bacterio)chlorophyll-specific branch of the porphyrin biosynthetic pathway	3.66	3.58
	D3H54_RS 30090		transposase	Transposase	1.08	Undetected
	D3H54_RS 30095	*cbiET/ cobL*	precorrin-6y C5,15-methyltransferase	Cobalamin biosynthesis	2.15	0.67
	D3H54_RS 30100	*bluB*	5,6-dimethylbenzimidazole synthase	Cobalamin biosynthesis	6.10	4.48
	D3H54_RS 30105	*cbiF/ cobM*	cobalt-precorrin-4/precorrin-4 C(11)-methyltransferase	Cobalamin biosynthesis	6.91	5.49
	D3H54_RS 30110	*cobIJ*	precorrin-2 C(20)-methyltransferase	Cobalamin biosynthesis	5.98	5.00
	D3H54_RS 30115	*cbiC*	precorrin-8X methylmutase	Cobalamin biosynthesis	6.77	4.86
	D3H54_RS 30120	*ibcL*	acetyl-CoA C-acetyltransferase (PhaA)	Transforms acetoacetyl-CoA into acetyl-CoA	5.54	4.84
	D3H54_RS 30125	*cobA/ pduO/ btuR*	cob(I)yrinic acid a,c-diamide adenosyltransferase	Cobalamin biosynthesis	6.42	5.92
	D3H54_RS 30130	*cobA*	uroporphyrinogen-III C-methyltransferase	Cobalamin biosynthesis	6.04	5.31

	Locus tag ID	Gene Name	NCBI RefSeq Gene Annotation	Predicted Function	log_2 FC RNA	log_2 FC Protein
	D3H54_RS 30135	*ibcM*	cobalamin B12-binding domain-containing protein (HcmB)	Transforms 2-hydroxyisobutyryl-CoA into 3-hydroxybutyryl-CoA	6.39	6.38
	D3H54_RS 30140	*ibcN*	phenylacetate--CoA ligase family protein (HCL)	Transforms 2-hydroxyisobutyrate into 2-hydroxyisobutyryl-CoA	7.61	5.52
	D3H54_RS 30145	*ibcO*	methylmalonyl Co-A mutase-associated GTPase (MeaB)	Mutase protection and stimulation. Transforms 2-hydroxyisobutyryl-CoA into 3-hydroxybutyryl-CoA	8.92	6.40
	D3H54_RS 30150	*ibcP*	methylmalonyl-CoA mutase family protein (HcmA)	Transforms 2-hydroxyisobutyryl-CoA into 3-hydroxybutyryl-CoA	7.92	6.32
	D3H54_RS 30155		FadR/GntR family transcriptional regulator	Transcriptional regulator	4.60	1.73
	D3H54_RS 30160	*ilvC*	ketol-acid reductoisomerase	BCAA biosynthesis	6.23	5.96
	D3H54_RS 32275		MHS family MFS transporter	Membrane transporter	6.19	3.58
	D3H54_RS 30170	*ibcQ*	3-hydroxybutyryl-CoA dehydrogenase (FadB)	Transforms 3-hydroxybutyryl-CoA into acetoacetyl-CoA	5.74	5.30
	D3H54_RS 32280		histidine phosphatase family protein	Unknown	6.86	1.49
	D3H54_RS 30180		thiamine pyrophosphate-binding protein/TPP lyase	Potential 2-hydroxyacyl-CoA lyase	5.26	5.04
	D3H54_RS 30185		AAA family ATPase	Transposase	1.91	Undetected
	D3H54_RS 30190		tyrosine-type recombinase/integrase	Transposase	-0.23	Undetected

	Locus tag ID	Gene Name	NCBI RefSeq Gene Annotation	Predicted Function	log_2 FC RNA	log_2 FC Protein
	D3H54_RS 30195		helix-turn-helix domain-containing protein	Transcriptional regulator	0.06	-0.17
	D3H54_RS 30200		tyrosine-type recombinase/integrase	Transposase	1.07	-0.25
	D3H54_RS 30205		ATP-binding protein	Unknown	0.79	Undetected
	D3H54_RS 30210		Mu transposase domain-containing protein	Transposase	0.86	Undetected
	D3H54_RS 30215		IS256 family transposase	Transposase	1.18	Undetected
	D3H54_RS 30220	*ibcA*	isoprene monooxygenase oxygenase subunit alpha	Subunit alpha. Transforms isobutylene into isobutylene oxide	4.22	3.53
	D3H54_RS 30225	*ibcB*	toluene-4-monooxygenase system B family protein	Subunit gamma. Transforms isobutylene into isobutylene oxide	4.33	3.93
	D3H54_RS 30230	*ibcC*	Rieske 2Fe-2S domain-containing protein	Ferrodoxin. Transforms isobutylene into isobutylene oxide	4.28	2.58
	D3H54_RS 30235	*ibcD*	MmoB/DmpM family protein	Coupling protein. Transforms isobutylene into isobutylene oxide	4.29	2.56
	D3H54_RS 30240	*ibcE*	toluene hydroxylase	Subunit beta. Transforms isobutylene into isobutylene oxide	4.16	3.84
	D3H54_RS 30245	*ibcF*	2Fe-2S iron-sulfur cluster-binding protein	Oxidoreductase. Transforms isobutylene into isobutylene oxide	4.05	3.28

	Locus tag ID	Gene Name	NCBI RefSeq Gene Annotation	Predicted Function	log_2 FC RNA	log_2 FC Protein
	D3H54_RS 30250	*ibcG*	aldehyde dehydrogenase family protein	Unknown	3.87	4.09
	D3H54_RS 30255	*ibcH*	GMC family oxidoreductase (MpdB)	Transforms 2-methyl-1,2-propanediol into 2-hydroxyisobutyraldehyde	4.23	3.48
	D3H54_RS 30260	*ibcI*	hypothetical protein	Unknown	4.24	2.87
	D3H54_RS 30265	*ibcJ*	aldehyde dehydrogenase family protein (MpdC)	Transforms 2-hydroxyisobutyraldehyde into 2-hydroxyisobutyrate	4.21	3.50
	D3H54_RS 30270	*ibcK*	alpha/beta fold hydrolase	Transforms isobutylene oxide into 2-methyl-1,2-propanediol	4.38	2.80
	D3H54_RS 32285		IS1634 family transposase	Transposase	Undetected	Undetected
	D3H54_RS 30280		2Fe-2S iron-sulfur cluster-binding protein	Group 6 SDIMO	1.17	0.30
	D3H54_RS 30285		MmoB/DmpM family protein	Group 6 SDIMO	1.28	0.11
	D3H54_RS 30290		aromatic/alkene monooxygenase hydroxylase subunit beta	Group 6 SDIMO	1.21	0.47
	D3H54_RS 30295		aromatic/alkene/methane monooxygenase hydroxylase/oxygenase subunit alpha	Group 6 SDIMO	1.52	0.62
	D3H54_RS 30300		Two-component system response regulator LuxR family	Two-component system regulator	1.61	0.78

	Locus tag ID	Gene Name	NCBI RefSeq Gene Annotation	Predicted Function	$\log_2$ FC RNA	$\log_2$ FC Protein
	D3H54_RS 30305		GAF domain-containing protein	Histidine sensor kinase	2.60	2.54

The selection criteria for considering significant up- or down- regulation satisfied the following criteria: $|\log_2 FC|$ value ≥ 1, adjusted p-value ≤ 0.05. All RNA-seq $\log_2$ fold changes listed have an FDR p-value < 0.05. All proteomic $\log_2$ fold changes listed have a p-value < 0.05 except for proteins not identified and D3H54_RS30200. *This protein was identified by mass spectrometry; however, it could not be distinguished from two other chromosomal peptide sequences and therefore is inconclusive.

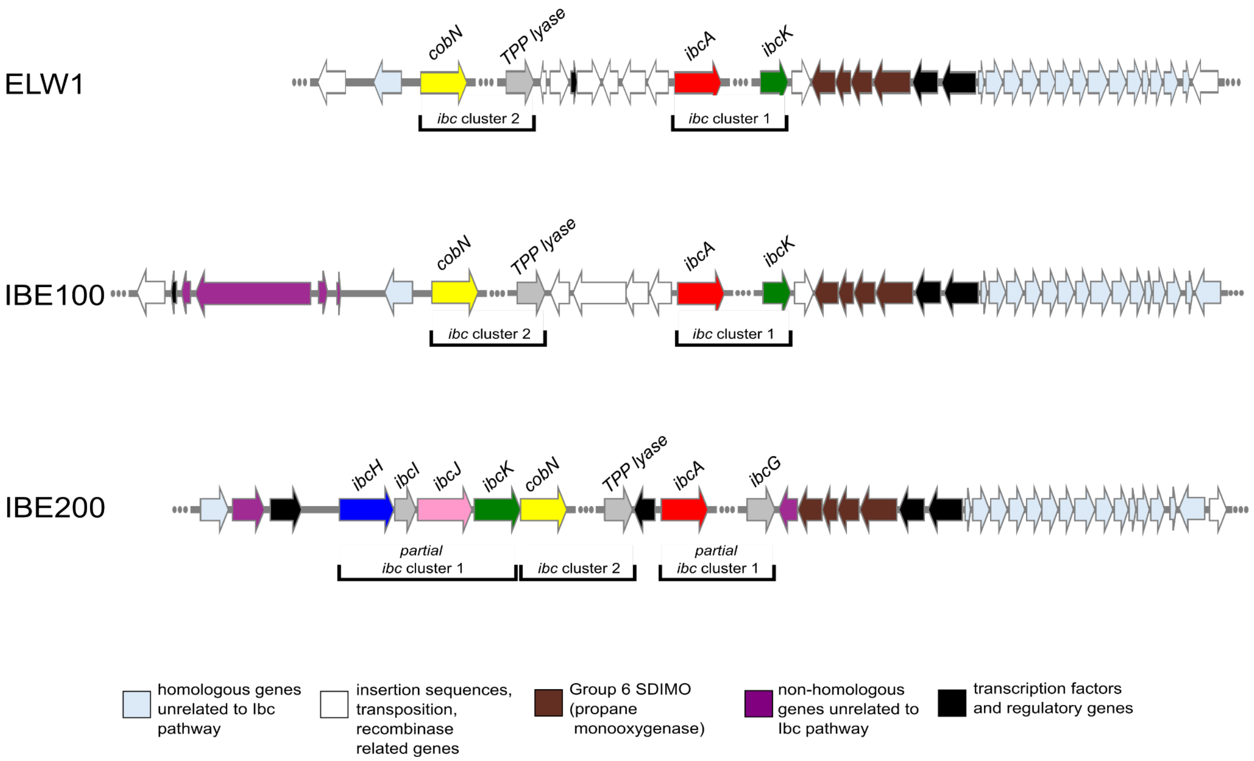

Figure S2. Genes flanking *ibc* cluster 1 and 2 of IB catabolizing bacteria.
Genes flanking the *ibc* gene clusters in *Mycolicibacterium* sp. ELW1, *Mycolicibacterium gadium* IBE100 and *Mycobacterium paragordonae* IBE200. Genes are colored to match homologous sequences unless specifically labeled for IBE200, in which cluster 1 is split. Black and white colored genes may or may not be homologous. Gene depictions are not to scale.

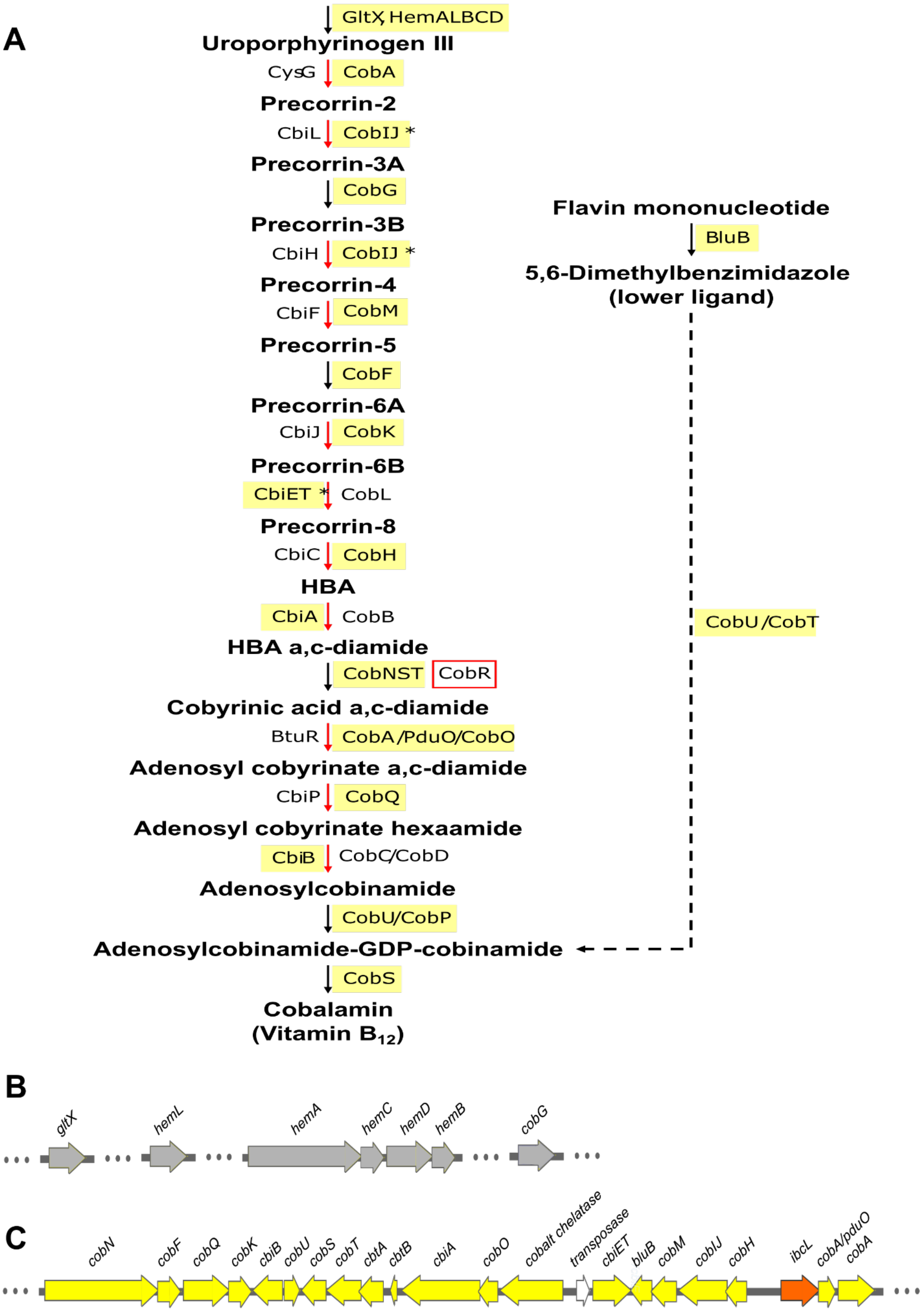
A
GltX, HemALBCD
Uroporphyrinogen III
CysG
CobA
Precorrin-2
CbiL
CobIJ *
Precorrin-3A
CobG
Precorrin-3B
CbiH
CobIJ *
Precorrin-4
CbiF
CobM
Precorrin-5
CobF
Precorrin-6A
CbiJ
CobK
Precorrin-6B
CbiET *
CobL
Precorrin-8
CbiC
CobH
HBA
CbiA
CobB
HBA a,c-diamide
CobNST
CobR
Cobyrinic acid a,c-diamide
BtuR
CobA/PduO/CobO
Adenosyl cobyrinate a,c-diamide
CbiP
CobQ
Adenosyl cobyrinate hexaamide
CbiB
CobC/CobD
Adenosylcobinamide
CobU/CobP
Adenosylcobinamide-GDP-cobinamide
CobS
Cobalamin
(Vitamin B12)
Flavin mononucleotide
BluB
5,6-Dimethylbenzimidazole
(lower ligand)
CobU/CobT
B
gltX
hemL
hemA
hemC
hemD
hemB
cobG
C
cobN
cobF
cobQ
cobK
cbiB
cobU
cobS
cobT
cbtA
cbtB
cbiA
cobO
cobalt chelatase
transposase
cbiET
bluB
cobM
cobIJ
cobH
ibcL
cobA/pduO
cobA

Figure S3. The aerobic cobalamin biosynthesis pathway and predicted cobalamin biosynthesis genes in *Mycolicibacterium* sp. ELW1.

A) The aerobic cobalamin biosynthesis pathway. Yellow boxes indicate homologous gene sequences are found in ELW1. Red arrows indicate sequence homology and functional equivalency between aerobic and anaerobic pathway enzymes. * represents a likely bifunctional enzyme based on sequence homology. This is especially true for *cbiET* which can be found as a bifunctional enzyme (2), however, *cobIJ* has no direct evidence, only gene homology predictions aligning to both *cobI* and *cobJ*. The red box indicates a gene not found by gene annotation in ELW1. While the gene *cobR* has been elucidated in the aerobic pathway, no dedicated enzyme for cobalt reduction at this step in the anaerobic cobalamin biosynthesis pathway has been identified (3). It is unknown if this is a necessary enzyme for ELW1. Functional annotations of the genes of interest were corroborated using BLASTp (NCBI). Pathway adapted from Lu et al. (4) and Shelton et al. (5). **B**) Relevant ELW1 chromosomal cobalamin biosynthesis genes not found on the plasmid pELW1-1. **C**) pELW1-1 *ibc* cluster 2 cobalamin biosynthesis genes.

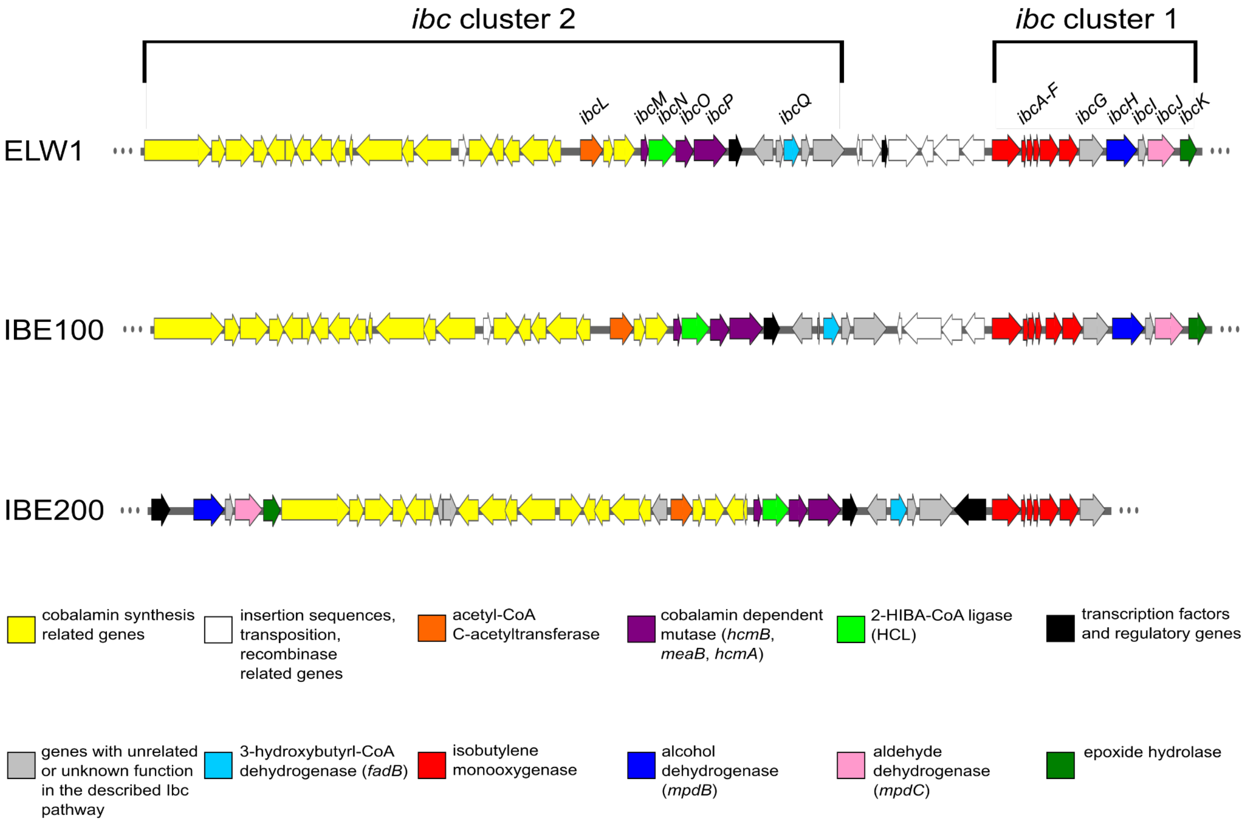

Figure S4. *ibc* clusters in pELW1-1 and in the contigs of IBE100 and IBE200. The *ibc* gene clusters in *Mycolicibacterium* sp. ELW1, *Mycolicibacterium gadium* IBE100 and *Mycobacterium paragordonae* IBE200. Genes are colored to match homologous sequences. Black and white colored genes may or may not be homologous. Gene depictions are not to scale.

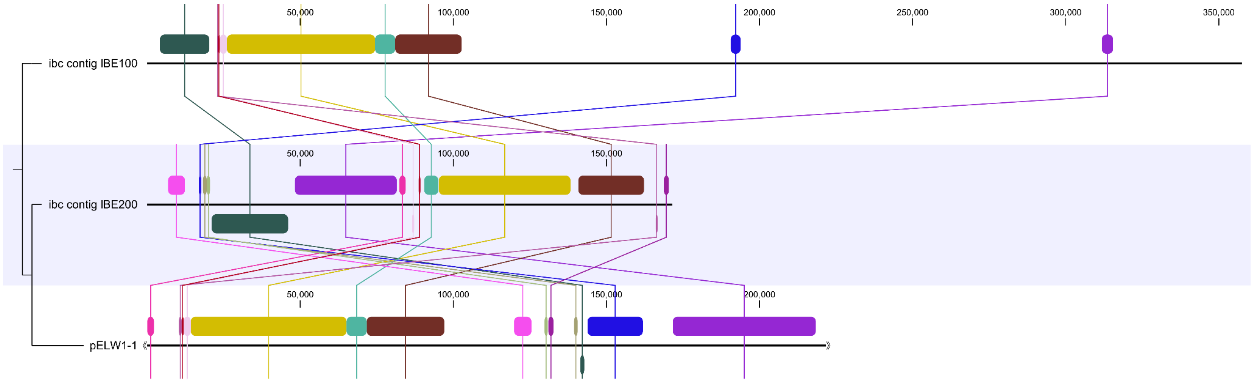

Figure S5. Sequence similarities of pELW1-1 and the *ibc* cluster-containing contigs in IBE100 and IBE200.

The taxonomic and genomic alignment comparison between pELW1-1 and the *ibc* cluster-containing contigs from IBE100 and IBE200. *ibc* Cluster 1 is indicated by the brown and light blue-green-colored blocks. *ibc* Cluster 2 is represented by the gold-colored block. Alignments were performed using CLC Genomics Workbench v23.0.4 (QIAGEN) Whole Genome Alignment tool with default settings. The results show a phylogenetic comparison of the nucleotide sequences between these three species and their *ibc* cluster containing contigs. The colored blocks represent significant nucleotide sequence homology. There is greater similarity between pELW1-1 and the IBE200 contig compared to the IBE100 contig despite differences in gene organization within the *ibc* clusters.

Table S4. Quantitative proteomic analysis of the most abundant proteins (10) identified by ABL and SDS-PAGE/NIR from soluble fractions of IB-grown ELW1 cells.

Band ID	Total proteins identified	Protein ID [a]	Gene description	Coverage (%)	# PSMs [b]	kDa	Abundance (Normalized)
~58 kDa (Band 01)	648 proteins identified (16 contaminants, primarily keratins)	QEN17701	*ibcA*	78	635	58	8.50E+10
		QEN17570	*ibcJ*	71	172	52.1	1.50E+10
		QEN13286	ATP synthase subunit beta	94	204	51.7	1.25E+10
		QEN13010	Fumarate hydratase	75	178	49.9	1.12E+10
		QEN12456	aldehyde dehydrogenase	76	172	54.8	1.02E+10
		QEN17568	*ibcH*	88	152	59.8	9.35E+09
		QEN12438	Chaperonin GroEL	84	138	56.5	8.14E+09
		QEN13004	Serine hydroxymethyl-transferase	87	152	52	7.33E+09
		QEN12190	NAD-dependent succinate-semialdehyde dehydrogenase	92	115	47.8	4.65E+09
		QEN15565	Adenosyl-homocysteinase	75	72	53.3	2.83E+09

Band ID	Total proteins identified	Protein ID [a]	Gene description	Coverage (%)	# PSMs [b]	kDa	Abundance (Normalized)
~68 kDa (Band 02)	484 proteins identified (16 contaminants, primarily keratins)	QEN17568	*ibcH*	92	793	59.8	1.04E+11
		QEN12438	Chaperonin GroEL	97	612	56.5	7.93E+10
		QEN13284	ATP synthase subunit alpha	81	212	58.7	1.91E+10
		QEN13959	30S Ribosomal protein	83	159	53.4	6.33E+09
		QEN15587	Acyl-CoA carboxylase subunit beta	76	145	58.4	4.82E+09
		QEN12120	Acyl-CoA dehydrogenase	76	118	66.5	4.69E+09
		QEN12098	Phosphoenol-pyruvate carboxykinase	82	157	66.8	4.36E+09
		QEN14586	Methylmalonyl-CoA mutase	67	74	64.1	2.71E+09
		QEN12283	Chaperonin DnaK	86	117	66.1	2.37E+09
		QEN17551	*ibcP*	65	111	64	2.29E+09

[a] Green highlights represent plasmid-encoded proteins. Protein ID is from the UniProt database. The table does not include identified contaminants.

[b] PSM (peptide spectrum matches).

Table S5. Gene expression levels comparing predicted plasmid-borne cobalamin biosynthesis genes to chromosomal homologs during growth on isobutylene.

Gene Name	pELW1-1 locus ID	Log_2 FC RNA	Log_2 FC Protein	Chromosome locus ID	Chromosome Protein ID	Log_2 FC RNA	Log_2 FC Protein
cobN	D3H54_RS 30025	2.91	1.71	D3H54_RS15545	QEN14468.1	0.59	0.04
cobF	D3H54_RS 30030	2.6	2.48	D3H54_RS23790	QEN15900.1	-0.24	-0.07
cobQ	D3H54_RS 30035	2.38	1.81	D3H54_RS18885	QEN15058.1	0.14	0.16
cobK/ cbiJ	D3H54_RS 30040	2.24	2.25	D3H54_RS15605	QEN14478.1	0.19	0.06
cbiB	D3H54_RS 30045	1.74	1.22	D3H54_RS11445	WP_149379135.1	0.01	0.08
cobU	D3H54_RS 30050	1.19	0.14	D3H54_RS11695	QEN17157.1	0.20	-0.06
cobS	D3H54_RS 30055	1.85	1.97	D3H54_RS11685	WP_149379175.1	0.23	0.33
cobT	D3H54_RS 30060	1.86	1.71	D3H54_RS11690	WP_168215064.1	0.16	0.01
cbtA	D3H54_RS 30065	1.91	0.97	D3H54_RS05595<br>D3H54_RS26145	WP_149378220.1<br>WP_149382507.1	-0.26<br>0.79	NA<br>1.14
cbtB	D3H54_RS 30070	2.03	NA*	D3H54_RS05590<br>D3H54_RS26150	WP_149378219.1<br>WP_36341779.1	-0.34<br>0.90	NA*
cbiA/ cobB	D3H54_RS 30075	3.36	1.69	D3H54_RS18685	WP_149380317.1	0.31	0.16
cobO	D3H54_RS 30080	3.57	3.24	D3H54_RS18690	WP_149380318.1	0.27	0.22
cbiET/ cobL	D3H54_RS 30095	2.15	0.67	D3H54_RS15615	WP_149379806.1	-0.20	0.03
bluB	D3H54_RS 30100	6.1	4.48	D3H54_RS21205	WP_149380898.1	-0.05	-0.39
cobM/ cbiF	D3H54_RS 30105	6.91	5.49	D3H54_RS15610	WP_149379805.1	-0.06	0.01
cobIJ	D3H54_RS 30110	5.98	5	D3H54_RS15595	WP_149379802.1	0.14	-0.13
cobH/ cbiC	D3H54_RS 30115	6.77	4.86	D3H54_RS15590	WP_149379801.1	0.17	0.24

Gene Name	pELW1-1 locus ID	Log_2 FC RNA	Log_2 FC Protein	Chromosome locus ID	Chromosome Protein ID	Log_2 FC RNA	Log_2 FC Protein
cobA/ PduO/ btuR	D3H54_RS 30125	6.42	5.92	D3H54_RS18690<br>D3H54_RS08495	WP_149380318.1<br>WP_149378670.1	0.27<br>0.31	0.22 -0.40
cobA	D3H54_RS 30130	6.04	5.31	D3H54_RS18680	WP_149380316.1	0.20	-0.25

Table S6. Primers for pELW1-1 PCR verification.

Fragment description	Primer direction	Sequence	Tm (℃)	GC (%)	Fragment size (bp)
cobN	Forward	CTCATCCAGACC CAGATCGC	57	60	1089
	Reverse	CTGGACTCTGC GACCCAAAT	57	55	
PPE domain containing protein	Forward	CAGAACCTCGTT GACACCGA	57	55	1235
	Reverse	TCGATGTTGGTG AAGTCCGG	57	55	
Cytochrome C biogenesis protein	Forward	GGGTACCGCCT TTACAACCA	57	55	978
	Reverse	ACTGGCTGCCA CAACTACTC	57	55	

Table S7. RNA-seq sequencing and mapping statistics for IB-grown vs fructose-grown cells of *Mycolicibacterium* sp. ELW1.

Sample Name [a]	Total number of reads	Reads mapped in pairs (%)	Reads not mapped (%)
IB1	229,858,486	97.72	1.67
IB2	172,721,708	98.08	1.27
IB3	270,752,406	98.09	1.36
IB5	295,565,146	97.94	1.47
F1	155,393,554	97.93	1.37
F2	101,263,470	97.31	1.97
F3	165,974,000	98.01	1.29
F4	164,545,494	97.85	1.44
F5	183,036,174	97.80	1.45

[a] IB = biological replicates of IB-grown cells of ELW1. F = biological replicates of fructose-grown cells of ELW1.

Table S8: CLC RNA-seq pipeline

CLC Genomics Workbench v23.0.4 (QIAGEN) RNA-seq pipeline was used.
Importing Illumina reads from NovaSeq 6000 data with CLC Genomics Workbench v23.0.4 (QIAGEN)

1. Illumina High-Throughput Sequencing Import
    a. Files per sample
        i. R1
        ii. R2
2. General Options (checked)
    a. Paired reads
    b. Discard read names
    c. Discard quality scores
3. Paired read information (checked)
    a. Paired-read (forward-reverse)
    b. Default min distance 1 and max distance 1000
4. Illumina options (checked)
    a. Remove failed reads
    b. Quality scores
        i. NCBI/Sanger or Illumina pipeline 1.8 and later
5. Result handling
    a. Save
    b. Create subfolders
    c. Create log

QC for Sequencing Reads

1. Prepare Sequencing Data
    a. QC for Sequencing Reads
2. Result handling
    a. Create graphical report (checked)
    b. Create supplementary report (checked)
    c. Save

Trimming reads

1. Prepare Sequencing data
    a. Trim reads
        i. Select imported paired reads
    b. Quality trimming (checked)
        i. Trim using quality scores
            1. Quality limit 0.05
        ii. Trim ambiguous nucleotides
            1. Max number = 2
    c. Adapter Trimming (checked)
        i. Automatic read-through adapter trimming
    d. Homopolymer trimming
        i. None checked
    e. Sequence trimming

    i. Defaults
  f. Sequence filtering
    i. Defaults
  g. Result handling (checked)
    i. Create report
    ii. Save
    iii. Crate log

Read Mapping and Small RNA Analysis

1. RNA-Seq Tools
    a. RNA-Seq Analysis (**must use version 23.0.4 or beyond**)
        i. Select paired, trimmed reads from previous section
    b. Reference Settings (checked)
        i. Genome annotated with genes only
            1. Ref sequence ELW1 Reference Genome (Genome)
                a. From NCBI reference download tool on CLC
                b. NZ_CP032155.1 and NZ_CP032156.1 used
            2. Gene track
                a. Created with the ELW1 reference genome using CLC
            3. Do not use spike-in controls
    c. Mapping settings
        i. All default including Maximum number of hits for a read
            1. This was set to 10
                a. 2 may have worked just as well
                b. Previous settings of 1 resulted in unmapped reads due to duplicate copies of genes in genome, and the subsequent discarding of them by the program
    d. Expression Settings
        i. Strand setting
            1. Both
        ii. Library type setting
            1. Bulk
        iii. Expression level
            1. RPKM
    e. Output settings
        i. Fusion gene table settings
            1. 5
    f. Results Handling (checked)
        i. Create reads track
        ii. Create report
        iii. Create fusion gene table
        iv. Create list of unmapped reads

Quality Control

1. QC for Read Mapping
    a. Select mapped reads

2. Result handling
    a. Output options
        i. Create separate table with statistics for each mapping (checked)
        ii. Save

Differential expression in two groups

1. Select expression sample
    a. Selected all of IB samples using the (GE) track
2. Control group expressions
    a. Selected all of the Fructose samples
3. Configure normalization method (checked)
    a. Whole transcriptome RNA-Seq
    b. All other defaults
4. Configure filtering and outliers (checked)
    a. Filter on average expression for FDR correction
5. Result handling (checked)
    a. Save
    b. Create log

When performed in this manner with reference genomes NZ_CP032155.1 and NZ_CP032156.1, there are two reads for *meaB* (D3H54_RS30145 and D3H54_RS32025). This is likely due to a sequencing error (deletion of a G) in our initial sequenced genome at position 42,936 in NZ_CP032156.1. RNA-seq data suggests the frameshift seen at that location is false, with the correct base observed in the RNA-seq data. The 2024 sequencing data do not show this deletion and does not show two reads. Given the similarity between the $\log_2$ fold changes of D3H54_RS30145 and D3H54_RS32025, we interpret this as a single transcribed gene in the differential expression analysis. There were other genes with frameshifts and incomplete coding sequences that contributed to partial transcripts named to locus IDs that do not match the accession sequences for NZ_CP032155.1 and NZ_CP032156.1. These errors are likely due to lower sequence quality for the ELW1 2019 sequence data; they are not present in the ONT + Illumina 2024 data which was of higher quality. However, due to the proteomic profiling using the initial sequence data CP032155.1 and CP032156.1 (NZ_CP032155.1 and NZ_CP032156.1) as reference, we used the same references for the initial RNA-seq analysis.

REFERENCES

1. Camacho C, Coulouris G, Avagyan V, Ma N, Papadopoulos J, Bealer K, Madden TL. 2009. BLAST+: architecture and applications. BMC Bioinformatics 10:421.

2. Moore SJ, Lawrence AD, Biedendieck R, Deery E, Frank S, Howard MJ, Rigby SEJ, Warren MJ. 2013. Elucidation of the anaerobic pathway for the corrin component of cobalamin (vitamin B_{12}). Proc Natl Acad Sci 110:14906–14911.

3. Lawrence AD, Deery E, McLean KJ, Munro AW, Pickersgill RW, Rigby SEJ, Warren MJ. 2008. Identification, characterization, and structure/function analysis of a corrin reductase involved in adenosylcobalamin biosynthesis. J Biol Chem 283:10813–10821.

4. Lu X, Heal KR, Ingalls AE, Doxey AC, Neufeld JD. 2020. Metagenomic and chemical characterization of soil cobalamin production. ISME J 14:53–66.

5. Shelton AN, Seth EC, Mok KC, Han AW, Jackson SN, Haft DR, Taga ME. 2019. Uneven distribution of cobamide biosynthesis and dependence in bacteria predicted by comparative genomics. ISME J 13:789–804.
